# *Desulfovibrio vulgaris* interacts with novel gut epithelial immune receptor LRRC19 and exacerbates colitis

**DOI:** 10.1186/s40168-023-01722-8

**Published:** 2024-01-03

**Authors:** Runxiang Xie, Yu Gu, Mengfan Li, Lingfeng Li, Yunwei Yang, Yue Sun, Bingqian Zhou, Tianyu Liu, Sinan Wang, Wentian Liu, Rongcun Yang, Xiaomin Su, Weilong Zhong, Bangmao Wang, Hailong Cao

**Affiliations:** 1 Department of Gastroenterology and Hepatology, Tianjin Medical University General Hospital, Tianjin Institute of Digestive Diseases, Tianjin Key Laboratory of Digestive Diseases, Tianjin, China; 2 Department of Immunology, Nankai University School of Medicine, Nankai University, Tianjin, China

**Keywords:** Ulcerative colitis, Flagellin, Leucine-rich repeat C19, Typhaneoside

## Abstract

**Background:**

The overgrowth of *Desulfovibrio*, an inflammation promoting flagellated bacteria, has been found in ulcerative colitis (UC) patients. However, the molecular mechanism in promoting colitis remains unestablished.

**Methods:**

The relative abundance *Desulfovibrio vulgaris* (*D. vulgaris*) in stool samples of UC patients was detected. Mice were treated with dextran sulfate sodium to induce colitis with or without administration of *D. vulgaris* or *D. vulgaris* flagellin (DVF), and the severity of colitis and the leucine-rich repeat containing 19 (LRRC19) signaling were assessed. The interaction between DVF and LRRC19 was identified by surface plasmon resonance and intestinal organoid culture. *Lrrc19*^*−/−*^ and *Tlr5*^*−/−*^ mice were used to investigate the indispensable role of LRRC19. Finally, the blockade of DVF-LRRC19 interaction was selected through virtual screening and the efficacy in colitis was assessed.

**Results:**

*D. vulgaris* was enriched in fecal samples of UC patients and was correlated with the disease severity. *D. vulgaris* or DVF treatment significantly exacerbated colitis in germ-free mice and conventional mice. Mechanistically, DVF could interact with LRRC19 (rather than TLR5) in colitis mice and organoids, and then induce the production of pro-inflammatory cytokines. *Lrrc19* knockdown blunted the severity of colitis. Furthermore, typhaneoside, a blockade of binding interfaces, blocked DVF-LRRC19 interaction and dramatically ameliorated DVF-induced colitis.

**Conclusions:**

*D. vulgaris* could promote colitis through DVF-LRRC19 interaction. Targeting DVF-LRRC19 interaction might be a new therapeutic strategy for UC therapy.

Video Abstract

**Supplementary Information:**

The online version contains supplementary material available at 10.1186/s40168-023-01722-8.

## Background

The incidence of inflammatory bowel disease (IBD) is rapidly increasing worldwide, likely be attributed to genetic and environmental factors [1]. These changes, associated with aberrant alterations in microbial composition and functionality, have been widely considered as major contributing factors for IBD [2]. It is generally accepted that high-fat diet (HFD)-induced gut dysbiosis could disrupt mucosal barrier, mediate intestinal inflammation, and ultimately promote the development of IBD [3]. However, the precise mechanism remains unclear. Sulfate-reducing bacteria (SRB) are anaerobic prokaryotes which inhabit the gastrointestinal tract of humans and animals [4]. Given their ability to produce hydrogen sulfide (H_2_S) by dissimilatory sulfate reduction, recent studies are increasingly focusing on the association between increased fecal levels of SRB and ulcerative colitis (UC) [5, 6]. Specifically, the most predominant constituents of SRB, *Desulfovibrio* spp., have been reported to be increased in crypt mucous gel of UC patients [4, 7]. Of interest, both current study and our previous study indicate that *Desulfovibrio* genus is the principal increased bacterial agent after HFD feeding, implying *Desulfovibrio* may be an important contributing factor in HFD-induced colitis [8–10]. However, the direct relationship between *Desulfovibrio* spp. and colitis remains largely unknown.

Pattern recognition receptors (PRRs) can recognize microbial pathogens and trigger the induction of pro-inflammatory cytokines [11]. Leucine-rich repeat domain is key component of PRRs such as Toll-like receptors (TLRs) [12]. Our previous study reported a novel leucine-rich repeat containing (LRRC) protein LRRC19, which is highly expressed in human and mouse intestinal epithelial cells [13]. The intracellular domain of LRRC19 contains no cytoplasmic Toll/interleukin 1 receptor (TIR) domain, which is distinct from the TLRs [14]. LRRC19 can recognize multiple TLR ligands such as flagellin and subsequently activate nuclear factor kappa-light-chain-enhancer of activated B cells (NF-κB) and mitogen-activated protein kinases (MAPK) pathways through TRAF2 (tumor necrosis factor receptor associated factor 2) and TRAF6 signaling pathways [15]. Considering the crucial role of LRRC19 in gut host-microbiota interaction, we sought to investigate whether *Desulfovibrio vulgaris* (*D. vulgaris*) or *D. vulgaris* flagellin (DVF) could interact with LRRC19 and exacerbate colitis.

In the present study, we confirmed that the abundance of *D. vulgaris* was increased in fecal samples of UC patients, which was paralleled by increased expression of LRRC19 in colonic mucosa. We demonstrated *D. vulgaris* administration promoted dextran sulfate sodium (DSS)-induced colitis in mouse model. This effect was mediated by interactions between DVF and LRRC19, which in turn initiated the TRAF6-mediated MAPK and NF-κB cascades, promoted the recruitment of immune cells, and increased the production of pro-inflammatory cytokines. Depletion of LRRC19 or blocking the DVF-LRRC19 interaction by typhaneoside, a flavonoid glycoside, significantly attenuated DVF-mediated intestinal inflammation.

## Methods

### Patients

Human stool samples were collected from UC patients and healthy controls recruited from General Hospital of Tianjin Medical University (Tianjin, China). The samples were snap-frozen and stored at − 80 °C. UC patients at the active stage (Mayo score ≥ 3) were diagnosed by radiology, endoscopy, and histology. Exclusion criteria included antibiotics, steroids, or probiotics use in the previous 3 months, intestinal infection, functional gastrointestinal disorders, short bowel syndrome or history of gastrointestinal surgery, malignant tumors, diabetes mellitus, pregnancy, generalized inflammation, active cardiovascular, renal or liver disease, and autoimmune disease. Age- and sex-matched healthy controls who had no current or recent use of antibiotics or probiotics within the past month were included. The general characteristics of the patients and healthy controls are presented in Supplementary Table S1, and the clinical characteristics of the included UC patients are presented in Supplementary Table S2.

### Bacterial strains and culture conditions

*D. vulgaris* (29579) was purchased from American Type Culture Collection (ATCC) and was maintained in Modified Baar’s Medium (ATCC Medium1249) under anaerobic conditions (80% N_2_, 10% H_2_, 10% CO_2_) at 30 °C.

### Desulfovibrio vulgaris quantification

The abundance of *D. vulgaris* in stool samples was quantified by quantitative PCR (qPCR) according to previously published protocols [16]. Total microbial DNA was extracted using the QIAamp DNA stool kit (QIAGEN, Germany). *D. vulgaris* quantitation was measured relative to the universal bacteria 16S, the primers are listed in Supplementary Table S3.

### Expression and purification of DVF in vitro

Recombinant DVF was expressed in *Escherichia coli* (*E. coli*) as described previously [17]. His-tagged DVF gene was cloned into the PSMART-I vector using the BamHI and XhoI restriction enzyme sites (Fig. 2A). Construct was verified by visualizing MluI and XhoI digested fragments on an agarose gel (Figure S1A) and transformed into *E. coli* BL21 (DE3). *E. coli* BL21 was cultured in Luria broth (LB) broth at 37 °C and 220 rpm shaking for 45 min and then subcultured in LB broth with kanamycin (50 µg/mL) at 37 °C overnight. At an OD600 of 1.0, the bacteria were harvested. After lysed by sonification, the supernatant was removed and the bacterial lysate was collected to purify the DVF protein. Purified DVF was identified by BIOTREE.

### Animal experiments

Female C57BL/6 mice (8 weeks of age) were housed with 5 mice per cage in the light- and temperature-controlled facility under specific pathogen-free circumstance. Acute experimental colitis was induced via administration of 2% dextran sulfate sodium (DSS, MP Biomedicals, molecular weight 35–50 kDa) in the drinking water for 7 days. DSS solution was replaced every day. Body weight and disease activity index (DAI, determined by body weight loss, occult blood, and stool consistency) were measured every day during the experimental period. To investigate the effects of *D. vulgaris* on colitis, mice were treated with an antibiotic cocktail in drinking water (0.1 g/l vancomycin, 0.2 g/l ampicillin, neomycin, and metronidazole) for 5 days [18], which was refreshed every day. *D. vulgaris* (ATCC 29579) was resuspended in sterile phosphate-buffered saline (PBS) containing 2.5% glycerol at 2.5 × 10^8^ CFU/ml. Mice were treated with 200 μl of PBS, bacteria, or DVF (2 μg) daily by oral gavage from 3 days before DSS treatment to sacrifice.

Female and male *Lrrc19*^*−*/*−*^ mice and *Tlr5*^*−*/*−*^ mice were generated by Model Animal Research Center of Nanjing University (Nanjing, China). Adult *Lrrc19*^*−*/*−*^ mice or *Tlr5*^*−*/*−*^ mice (8 weeks of age) were divided into 2 groups randomly and were treated with either DVF or PBS, induction of DSS colitis was performed as previously described.

### Protein–protein docking and molecular screening

The protein crystal structure of DVF and LRRC19 was generated using the SWISS-MODEL server and Iterative Threading ASSEmbly Refinement (I-TASSER) server [19, 20]. The protein docking was conducted by HEX software, and the docking parameters were defined on the basis of the protein surface structure and surface potential. According to the interaction interface of DVF-LRRC19 complex, small-molecule compounds were selected from the traditional Chinese medicine (TCM) database through high-throughput screening.

### Organoid culture

The organoid culture was performed in accordance to the protocol described previously [21]. In brief, organoids were generated from isolated crypts of the colon of colitis mice (C57/BL6 mice and *Lrrc19*^*−*/*−*^ mice) and then embedded into Matrigel (Corning, Corning, New York, USA). After that, organoids were kept in Organoid Growth Medium (STEMCELL Technologies) in the presence of R-Spondin, Noggin, and EGF (Proteintech). To investigate the effect of DVF on organoids, organoids were co-cultured with 1 μg DVF or PBS on 6-well plates. After 5 days of co-culture, organoid morphologies were recorded and then harvested for further experiments.

### Organoid immunostaining and imaging

The organoid immunostaining was performed based on established protocols [22]. Harvested organoids were fixed using 4% paraformaldehyde for 1 h, embedded in paraffin at room temperature, and then cut into 4-µm slices sections. After deparaffinization and hydration, the sections were blocked with 5% bovine serum albumin, prestaining of eosin was performed during dehydration. Organoids were incubated with primary antibody against LRRC19 (Abcam, ab106657; 1:200) overnight at 4 °C, followed by incubated with corresponding secondary antibody (Santa Cruz Biotechnology, Inc). Quantification of the intensity of LRRC19 staining was performed using Image-Pro Plus 6.0 software.

### Surface plasmon resonance binding assays

Surface plasmon resonance experiments were performed on a BIACORE 3000 biosensor system (GE Healthcare) according to the manufacturer’s instructions at 25 °C. DVF proteins were prepared as mentioned above. LRRC19 protein (Cat. No. D623707) was purchased from Sangon Biotechnology (Shanghai, China). To investigate binding of either DVF or typhaneoside to LRRC19, LRRC19 protein was immobilized onto a CM5 chip (GE Healthcare), which was activated using a 1:1 mixture of 1-ethyl-3-(3-dimethyllaminopropyl) carbodiimide (EDC) and N-hydroxysuccinimide (NHS) at a flow rate of 10 μL/min. Residual unoccupied active groups were blocked by 1 M ethanolamine hydrochloride-NaOH, pH 8.5. DVF protein was diluted in Tris buffered saline while typhaneoside was diluted in PBS, and then injected over the LRRC19 surface at a rate of 30 μL/min flow rate. At least 5 different concentrations of DVF protein or typhaneoside were injected for each experiment. The duration of protein binding time was set to 180 s, after which the running buffer was injected at the same rate for 300 s. The sensor chip surface was regenerated by treating with 5 mM NaOH for 60 s after each cycle. The binding kinetics was processed and calculated by BIAevaluation software.

### Microscale thermophoresis

Microscale thermophoresis (MST) was conducted using a Monolith NT.115 (NanoTemper Technologies, Monolith, Germany). LRRC19 protein was labeled using a NT647 fluorescence dye (NanoTemper Technologies, Germany), by which 20 µM of protein was incubated with 60 µM dye solution at room temperature for 30 min. DVF protein or typhaneoside was dissolved in the MST buffer (50 mM Tris–HCl, pH 7.4; 150 mM NaCl; 10 mM MgCl_2_). The final reaction mixtures were loaded into NT.115 standard treated capillaries (NanoTemper Technologies) and repeated at least three times for each measurement. *K*_D_ was calculated using the MO Affinity Analysis v2.3 software.

### Typhaneoside treatments

Typhaneoside (CAS: 27,740–01-8, purity > 98.0%) was purchased from MCE company (New Jersey, United States). To examine the effects of typhaneoside on colitis, typhaneoside (40 mg/kg) was administered to two additional groups of mice (one group treated with PBS and the other treated with DVF) for 7 days together with DSS treatment. Body weight and disease activity index (DAI) were measured every day during the experimental period for DSS colitis.

### Statistical analysis

All statistical analyses were performed using GraphPad Prism 9.0 software. All data are presented as mean ± standard error of the mean. Statistical significance between two groups was determined by two-tailed unpaired Student’s *t* test when data were normally distributed, and nonparametric Mann–Whitney *U* test was used when data were not normally distributed. One-way ANOVA test was performed to compare the difference among multiple groups. *p* < 0.05 was considered as statistically significant.

## Results

### The abundance of D. vulgaris are enriched in the feces of UC patients

**Fig. 1:**
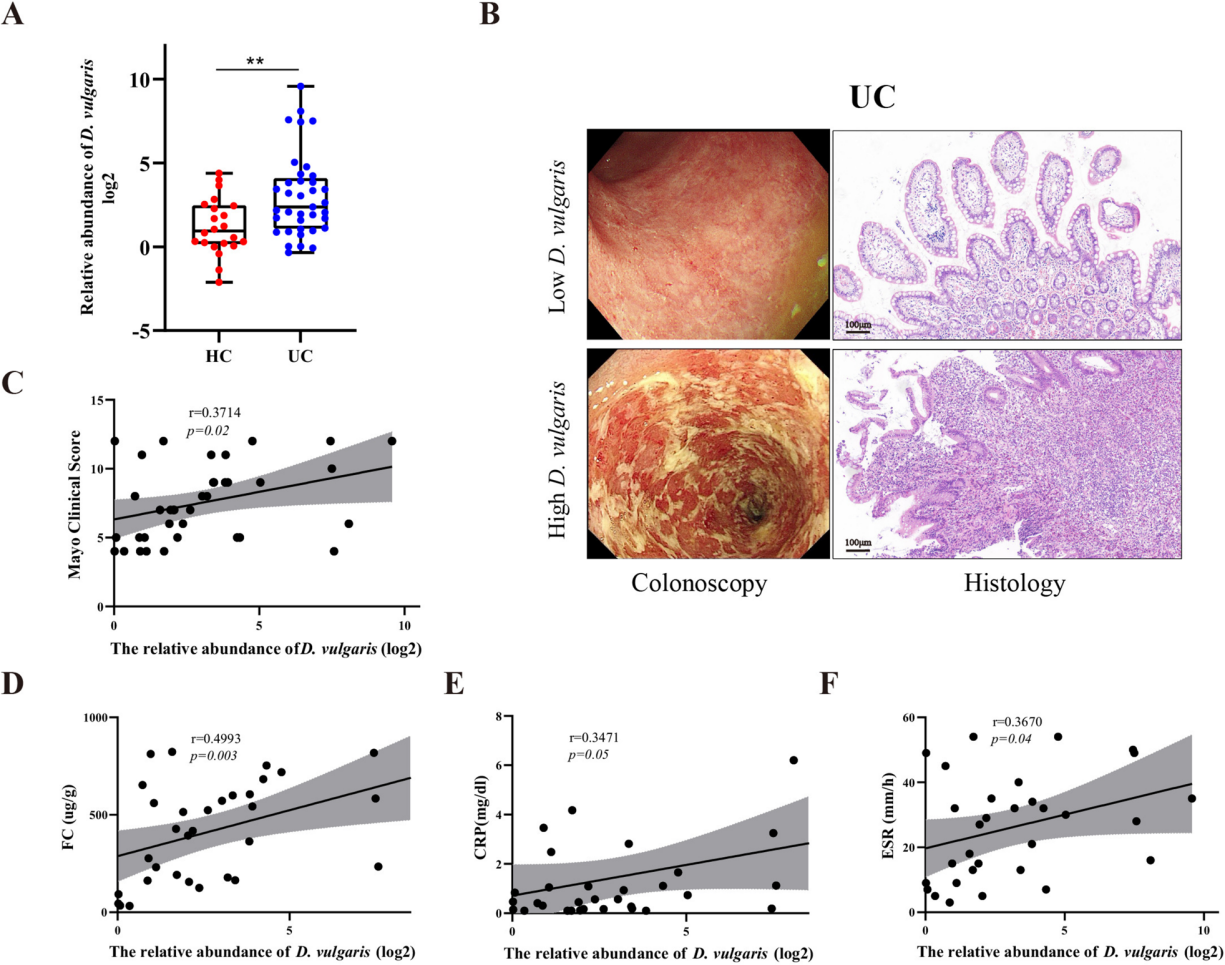
The abundance of *D. vulgaris* is enriched in the feces of UC patients and associated with disease severity. **A** The relative abundance of *D. vulgaris* in stool samples from UC patients (*n* = 37) and HC (*n* = 22). **B** Representative endoscopic images and histological pictures from UC patients with relatively high or low *D. vulgaris*. Scale bars, 100 µm. **C–F** Correlation analysis of the relationship between relative abundance of *D. vulgaris* and Mayo clinical score, *n* = 37 (**C**), FC, *n* = 33 (**D**), CRP, *n* = 33 (**E**), and ESR, *n* = 31 (**F**) in UC patients. All data are presented as mean ± SEM. **P < 0.01. Two-tailed Mann–Whitney *U* test in **A**; Spearman correlation analysis in **C–F**

To explore the abundance of *D. vulgaris*, we examined 59 stool samples from 37 UC patients and 22 healthy controls. The relative abundance of *D. vulgaris* were measured by quantitative PCR (qPCR). We found *D. vulgaris* abundance were significantly increased in the feces of UC patients (Fig. 1A). In addition, according to the results of qPCR, UC patients with relatively high *D. vulgaris* (relative abundance > 5.2) have more severe inflammation and mucosal ulceration as evaluated by colonoscopy and histopathology (Fig. 1B). Importantly, the relative abundance of *D. vulgaris* in feces were positively correlated with Mayo score (*r* = 0.3714, *p* = 0.02), fecal calprotectin levels (*r* = 0.4993, *p* = 0.003), C-reactive protein levels (*r* = 0.3471, *p* = 0.05), and erythrocyte sedimentation rate (*r* = 0.3670, *p* = 0.04) in UC patients (Fig. 1C–F). Taken together, our results suggest that *D. vulgaris* might be involved in the pathogenesis of UC.

### D. vulgaris promotes colitis in germ-free mice

To validate the effect of *D. vulgaris* on colitis, we gavaged germ-free mice with *D. vulgaris* (2.5 × 10^8^ colony-forming units (CFUs) per mouse) and then conducted DSS-induced colitis (Figure S2A). Compared with control group, mice in *D. vulgaris* group showed greater body weight loss and higher disease activity index (DAI) score during the experimental period (Figure S2B, C). After sacrifice, *D. vulgaris*-treated mice exhibited shorter colon length (Figure S2D). In line with these findings, representative histological examination results and histopathology scores also showed *D. vulgaris* significantly exacerbated the severity of colitis in comparison with the control group (Figure S2E). These results suggested that *D. vulgaris* promotes colitis in germ-free mice.

### D. vulgaris or DVF facilitates the experimental colitis in mice

**Fig. 2:**
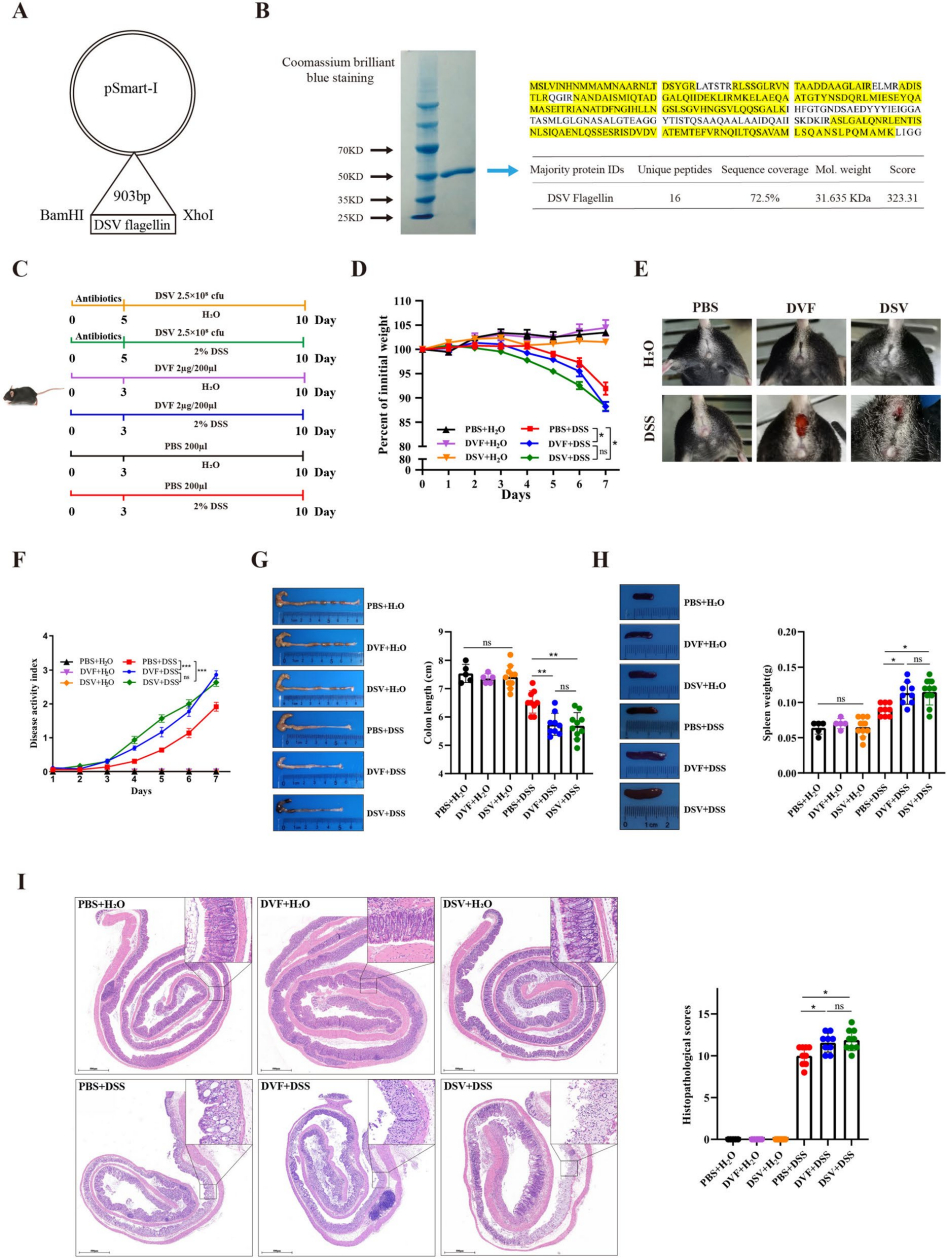
*D. vulgaris *or DVF facilitates the experimental colitis in mice.** A** His-tagged DVF (903 bp) was cloned into the pSmart-I vector using restriction endonuclease sites (BamHI/XhoI). **B** Coomassie Brilliant Blue staining of recombinant DVF (left panel) and the indicated band was excised and analyzed by mass spectrometry (right panel). **C** The experimental design of DSS mouse model. **D** Body weight was presented as a percentage of the initial weight. **E** Representative images of anal bleeding. **F–H** DAI (**F**), colon length (**G**), and spleen weight (**H**) were analyzed. (**I**) Representative histological images of colon tissues by H&E staining (left panel) and histopathological score (right panel). Scale bars, 500 µm. All data are presented as mean ± SEM. *P < 0.05, ** P < 0.01, *** P < 0.001, ns, not significant. Two-tailed Student’s *t* test in **D**; one-way ANOVA in **F–I**. DSV: *Desulfovibrio vulgaris*

It is widely accepted that increased flagellated microbiota, enhanced flagellar assembly, and elevated fecal flagellin levels are common features of dysbiosis in IBD patients [23, 24]. More specifically, a recent study has demonstrated that the flagellin of adherent-invasive *E. coli* (AIEC) are required in the AIEC-induced inflammation [25]. Hence, we presume that *D. vulgaris* might promote colitis via its flagellin. To investigate the role of DVF on colitis, the recombinant DVF was synthesized, purified, and identified (Fig. 2A, B; Figure S1A, B). Subsequently, *D. vulgaris* or DVF was administered to mice respectively, and then the colitis was inducted (Fig. 2C). The weight loss, anal bleeding, and DAI score were significantly increased in *D. vulgaris* or DVF-treated colitis mice (Fig. 2D-F). Compared with colitis mice treated by PBS, a marked shortening of the colon and larger spleens were observed in *D. vulgaris* or DVF group (Fig. 2G, H). Consistent with gross morphological indicators, colitis mice treated with *D. vulgaris* or DVF showed more severe histological damage, which was characterized by more crypt loss and infiltrating leucocytes (Fig. 2I). Interestingly, there were no significant differences in the colitis severity between *D. vulgaris* and DVF group. These results indicate that *D. vulgaris* could contribute to the development of colonic inflammation and the ability of *D. vulgaris* to induce exacerbation of colitis might be mediated by DVF.

### The effect of DVF on exacerbation of colitis is not through altering the gut microbiota

It has been reported that the constituent component of bacteria exerts their functions through altering the composition of the gut microbiota [26]. Here, to determine whether the impact of DVF on mice was via changing the gut microbiota, mice were gavaged with DVF for 10 days without DSS treatment and the feces were collected for 16S rRNA sequencing. Although there was a trend toward a decrease of alpha diversity (Chao1 and Shannon) index in DVF-treated group, the differences were not significant between two groups (Figure S3A). At the phylum level, no significant difference was found in the proportions of *Bacteroidetes*, *Firmicutes*, *Proteobacteria*, *Deferribacteres*, and *Actinobacteria* between two groups (Figure S3B). In addition, beta diversity also did not show significant differences between DVF-treated group and PBS-treated group using the Adonis test (*P* = 0.3, *R*^*2*^ = 0.125; Figure S3C). *LEfSe* results and LDA scores obtained from *LEfSe* analysis showed there were very few taxonomic differences between two groups (Figure S3D, E). Furthermore, functional pathways of the microbiome were predicted using PICRUSt2 package and annotated with KEGG database. The results showed the functional profile predictions of microbiota in two groups did not differ significantly between DVF group and PBS group (Figure S3F). Together, these findings implicate that DVF-mediated colonic inflammation in mice are not due to changes in gut microbiota composition.

### DVF leads to transcriptional activation of inflammatory genes

**Fig. 3:**
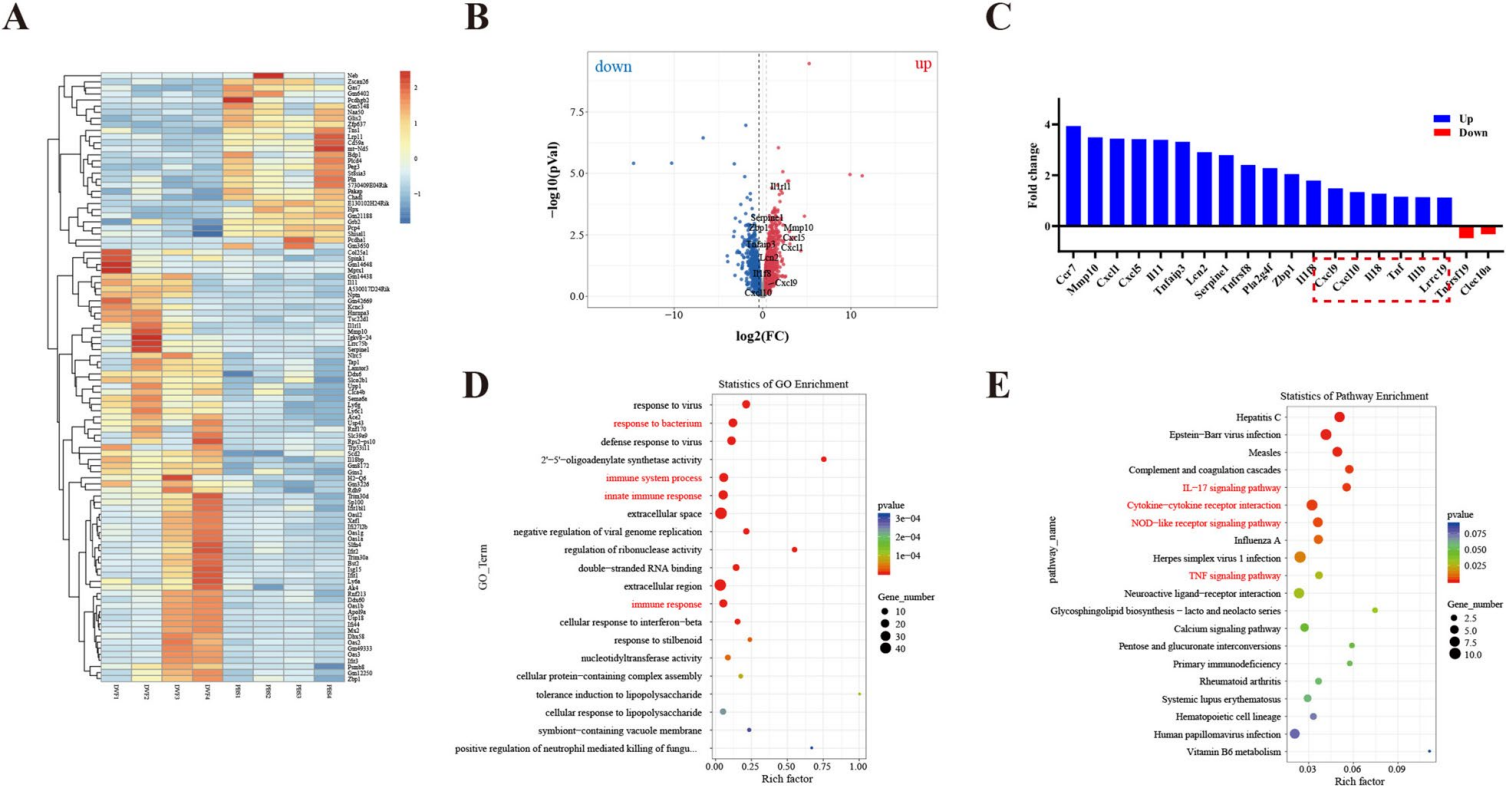
DVF leads to transcriptional activation of inflammatory genes. **A**,**B** Heatmap (**A**) and volcano plots (**B**) for RNA‐seq of colon tissue from DVF- or PBS-treated mice with colitis. **C** The fold change of selected chemokines, pro-inflammatory factors, and cytokines related to colitis. **D**,**E** GO analysis (**D**) and KEGG pathway analysis (**E**) of genes that are significantly upregulated in DVF-treated mice with colitis. All data are presented as mean ± SEM

Given DVF-mediated exacerbation of colitis was not due to alteration of gut microbiota, RNA‐seq of colon tissue from DVF or PBS-treated mice with colitis were performed to investigate the effect of DVF on colitis. Heatmap for differential gene expression between two groups was displayed in Fig. 3A. Volcano plots indicated that compared with PBS group, 512 genes are upregulated and 439 genes are downregulated in DVF group (Fig. 3B). Specifically, differential expressed genes (DEGs) related to colitis such as *Mmp10*, *Tnfaip3*, *Lcn2*, *Serpine1*, and *Pla2g4f* were upregulated in the colon tissue of colitis mice in DVF group (Fig. 3C). In addition, DEGs associated with the immune receptor (*Lrrc19*), cell chemotaxis (*Ccr7*, *Cxcl1*, *Cxcl5*, *Cxcl9*, and *Cxcl10*), and inflammatory response (*IL1r11*, *IL11*, *Tnf*, *IL1β*, and *IL18*) were also upregulated in the colonic tissue of colitis mice in DVF group (Fig. 3C). Based on the annotation in the GO database, these DEGs corresponded to immune system process, response to bacterium, and innate immune response (Fig. 3D). KEGG signaling pathway analysis also revealed these DEGs were involved in inflammatory pathways including IL − 17 signaling pathway, cytokine–cytokine receptor interactions, NOD − like receptor signaling pathway, and TNF signaling pathways (Fig. 3E). Validation of the enriched inflammatory genes were finally confirmed by real-time PCR analysis, western blotting, and ELISA (Figure S4A-C). Collectively, these results indicate that DVF can increase the expression of immune receptor LRRC19 and pro-inflammatory chemokines and cytokines.

### DVF does not induce the increased expression of TLR5

It is commonly claimed that bacterial flagellin can be recognized by TLR5; however, the structure of flagellin is highly variable even among members of the same bacterial family [27]. Based on the aforementioned RNA-seq results, we found the expression of *Tlr5* was not upregulated in colitis mice after DVF treatment (Figure S5A). This result was further validated by real-time PCR (Figure S5B). Moreover, the mRNA expression of *Tlr5* was not significantly upregulated in Caco2 cells after DVF stimulation (Figure S5C). These results suggest DVF does not induce the increased expression of TLR5.

Previous study has reported that TLR5 is not required for flagellin-mediated exacerbation of DSS colitis [28]. TLR5 gene expression was not upregulated in the mucosa of UC patients also suggesting TLR5 was independent of UC development (Figure S10A). In order to unequivocally specify the implications of TLR5 in DVF-induced exacerbation of DSS colitis, *Tlr5*^*−*/*−*^ mice were generated (Figure S5D) and were stimulated by DVF or not (Figure S5E). The weight loss and DAI score were significantly increased in DVF-treated colitis mice (Figure S5F, G). Compared with colitis mice treated by PBS, a marked shortening of the colon was observed in DVF group (Figure S5H). Consistent with gross morphological indicators, colitis mice treated with DVF showed more severe histological damage, which was characterized by more crypt loss and infiltrating leucocytes (Figure S5I). Altogether, these results demonstrate DVF-induced exacerbation of DSS colitis was not mediated by TLR5 signaling.

### DVF interacts with LRRC19

**Fig. 4:**
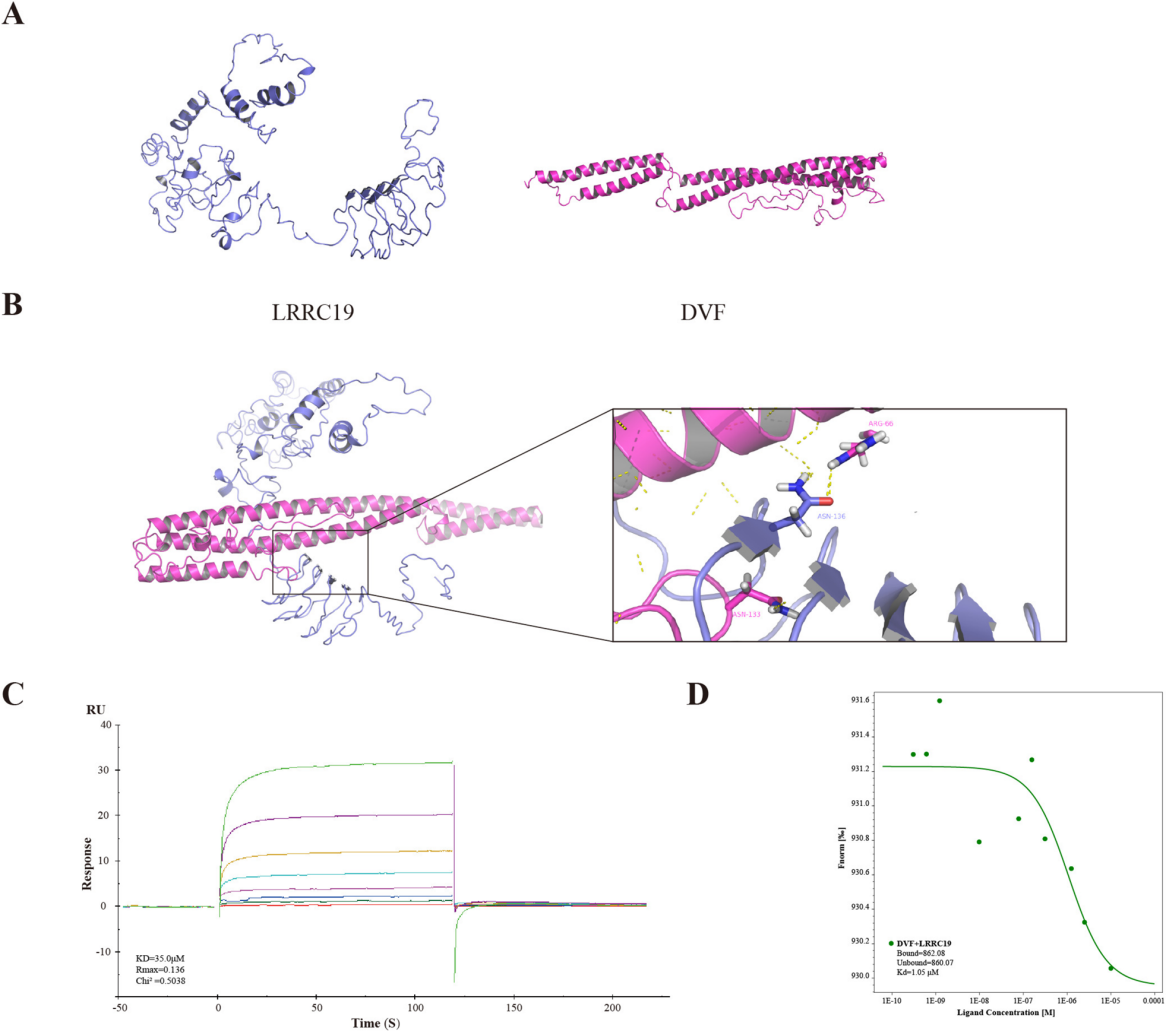
DVF interacts with LRRC19. **A** The crystal structure model of LRRC19 and DVF. **B** Protein–protein docking of DVF and LRRC19 and the interaction interface of amino acid in the binding site. **C**,**D** Biacore (**C**) and microscale thermophoresis (**D**) analysis of the interaction between DVF and LRRC19

Animal experiments have demonstrated LRRC19 may participate in the pathogenesis of colitis [13]. Since LRRC19 and its downstream pro-inflammatory chemokines were upregulated after DVF treatment in colitis, we hypothesized that DVF can activate LRRC19 and thus contribute to colonic inflammation. To verify this, the crystal structure of DVF and LRRC19 were generated using I-TASSER and SWISS-MODEL server because no 3D structure was available for the DVF and LRRC19 on the protein data bank (Fig. 4A; Figure S6A, B). Subsequently, the molecular docking was conducted and the results revealed significant interaction between DVF and LRRC19, and the amino acid on the binding sites of the interface was shown (Fig. 4B; Figure S6C). The docking results also suggest that DVF (rather than *Escherichia coli* flagellin or *Salmonella Typhimurium* flagellin) has a higher affinity for the LRRC19 (Supplementary Table S5).

To further confirm the interaction between DVF and LRRC19, SPR and microscale thermophoresis (MST) assay were conducted. SPR assay is widely recognized as a golden standard for characterizing protein–protein or small molecule–protein interactions [29]. The results of SPR demonstrated that the *K*_D_ for the DVF protein against LRRC19 was 35.03 μM, indicating the DVF-LRRC19 interaction was of high affinity (Fig. 4C). This result was also supported by MST experiments (Fig. 4D). Collectively, these data suggest that DVF could interact with LRRC19.

Our previous research has reported LRRC19 can recognize *E. coli* and activate NF-κB and MAPK cascades through inhibiting degradation of TRAF2 and increasing K63-linked ubiquitin on TRAF6 [15]. Based on the above findings, in order to prove that DVF can activate LRRC19 signaling in colonic epithelial cells, Caco2 and NCM460 cells were stimulated by DVF or not. After DVF treatment, the mRNA expression of *Lrrc19* and protein level of LRRC19 were significantly upregulated (Figure S7A, B). Concurrently, we found that K63-linked ubiquitin on TRAF6 in Caco2 cells was increased after DVF treatment (Figure S7C). However, DVF did not inhibit the degradation of TRAF2 (Figure S7C), suggesting that DVF interact with LRRC19 through combining with TRAF6 rather than TRAF2. We next determined whether DVF could activate NF-κB and MAPK cascades. Western blotting revealed that DVF increased the protein levels of P-p38, P-ERK, P-NF-κB p65, and P-IκBα in Caco2 cells (Figure S8A). In addition, we observed the mRNA expression of genes in the MAPK and NF-κB downstream pathway, including *Cxcl9*, *Cxcl10*, *IL1β*, and *IL8*, were also upregulated after DVF challenge (Figure S8B, C). All of these data showed that DVF could activate LRRC19 signaling, in turn mediate the activation of NF-κB and MAPK pathways through TRAF6-mediated K63-linked ubiquitin, and thus increase inflammatory gene expression.

To validate the pro-inflammatory effect of DVF in vitro, colon organoids derived from colitis mice (WT mice) were treated by DVF. The results showed that no significant difference was observed for the organoid per crypts ratio between 2 groups (Figure S9A). However, the organoids in DVF group proliferated and differentiated into more simple structures and consist a smaller number of crypt-like domains than the PBS group (Figure S9A). Moreover, we found DVF challenge significantly promoted apoptosis in the organoids and increased the expression of LRRC19 (Figure S9B, C).

Next, to demonstrate whether DVF-mediated activation of MAPK and NF-κB signaling depend on LRRC19, we further knocked down LRRC19 using LRRC19 small interfering RNA (siRNA) in Caco2 cells and treated these cells with DVF. We found that DVF-mediated activation of NF-κB and MAPK pathways were remarkably abolished by LRRC19 siRNA (Figure S8A). These findings confirm that DVF-induced activation of MAPK and NF-κB signaling was via interacting with LRRC19.

### DVF activates the LRRC19-MAPK/NF-κB pathway and promotes the recruitment of inflammatory immune cells in mice

To confirm the involvement of LRRC19 in UC, we measured the LRRC19 expression in colonic mucosa of UC patients. Compared with healthy controls, we found the protein levels of LRRC19 were significantly increased in the mucosa of UC patients (Figure S10B, C).

**Fig. 5:**
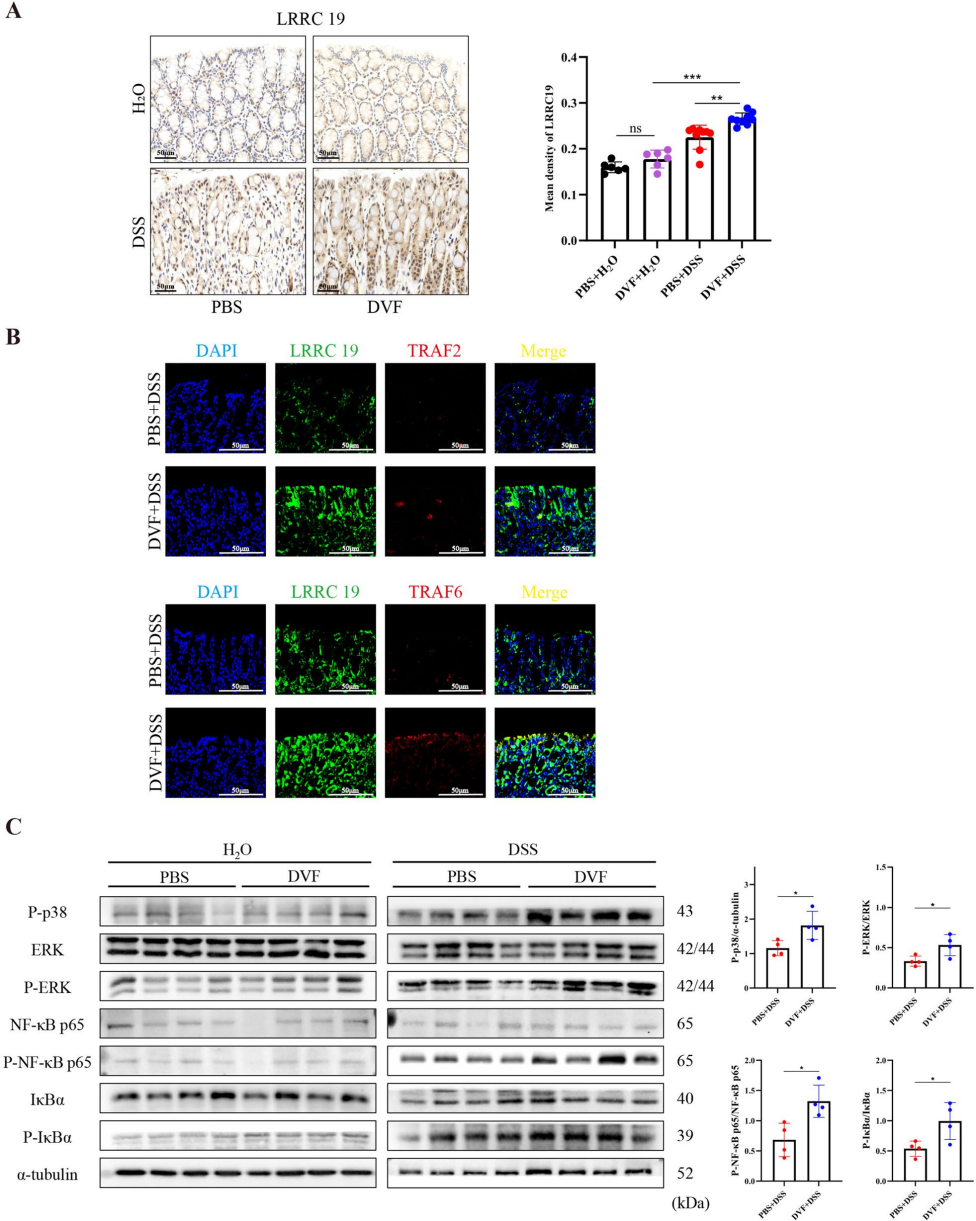
DVF activates the LRRC19-TRAF6-MAPK/NF-κB pathway in colitis mice.** A** IHC staining and quantitation of LRRC19 in the colonic mucosa of DVF- or PBS-treated mice. Scale bars, 50 µm. **B** Confocal fluorescent analyses of the interaction between LRRC19 and TRAF2 or TRAF6 in colitis mice. Scale bars, 50 µm. **C** The protein levels of P-p38, P-ERK, P-NF-κB p65, and P-IκBα of DVF- or PBS-treated mice were measured by western blotting, α-tubulin was used as loading control. Relative protein levels were quantified using the Imagelab. All data are presented as mean ± SEM. *P < 0.05, ** P < 0.01, *** P < 0.001, ns, not significant. One-way ANOVA in **A**, two-tailed Student’s *t* test in **C**

To validate DVF could activate LRRC19 signaling in vivo, we investigated the activation of LRRC19 pathway in DVF-treated colitis mice. Compared with PBS-treated colitis mice, the expression of LRRC19 was upregulated in DVF groups (Fig. 5A). Confocal immunostaining showed that the colocalization between LRRC19 and TRAF6 in DVF-treated colitis mice was highly visible, but colocalization of LRRC19 and TRAF2 was rarely observed (Fig. 5B). These results were consistent with our cell experiments in vitro. Besides, we found that the protein levels of P-p38, P-ERK, P-NF-κB p65, and P-IκBα were all upregulated in DVF groups (Fig. 5C). Integrating with the transcriptomic data, we found DVF were able to activate the LRRC19 and then direct interact with TRAF6, in turn mediate the activation of NF-κB and MAPK pathways and induce production of pro-inflammatory chemokines and cytokines in vivo.

Chemokines play an integral role in the recruitment of immune cells [30]. Accordingly, to address the effect of DVF on gut immune microenvironment, we performed multicolor flow cytometry. We observed the proportion of Ly6C^+^MHCII^+^ macrophages and CD11c^+^CD103^+^CD11b^+^ dendritic cells (DCs), which can stimulate the pro‐inflammatory responses, were significantly increased in the colon lamina propria (CLP) of DVF-treated colitis mice compared with PBS group (Figure S11A). Similar results were obtained in mesenteric lymph nodes (MLNs) and Peyer’s patches (PPs) (Figure S11B, C). These data suggest that DVF can recruit pro-inflammatory immune cells and provide a pro-inflammatory milieu, which may further facilitate the development of colitis.

### DVF does not aggravate colitis in LRRC19 knockout mice

**Fig. 6:**
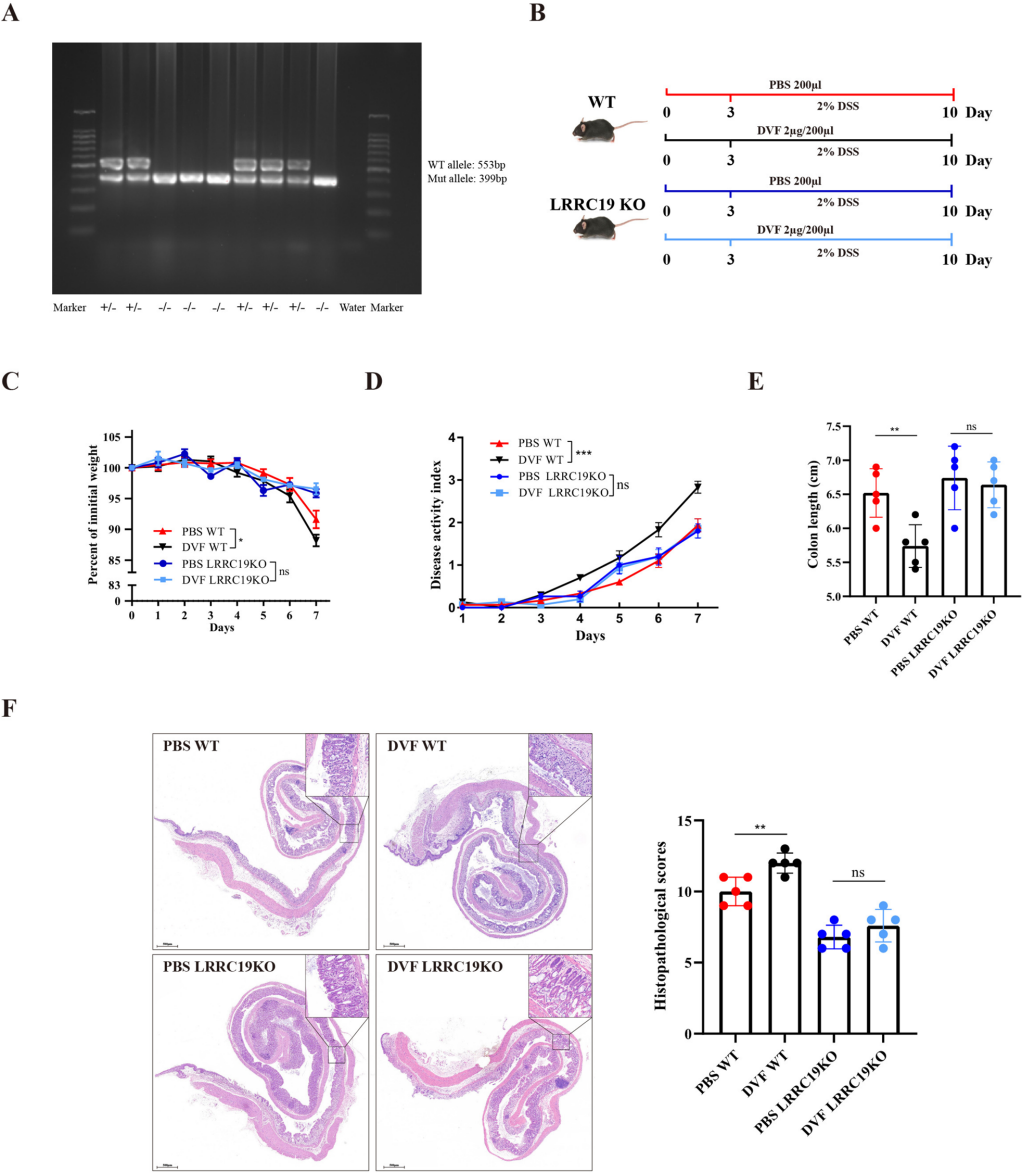
DVF does not aggravate colitis in LRRC19 knockout mice. **A** Mouse genotypes were determined by PCR using the indicated primers to detect wild-type and mutant alleles of *Lrrc19*. **B** The experimental design of DSS model in WT and *Lrrc19*^*−/−*^ mice. **C** Body weight was presented as a percentage of the initial weight. **D**,**E** DAI (**D**) and colon length (**E**) are shown. **F** Representative histological images of colon tissues by H&E staining (left; Scale bars, 500 µm) and histopathological score (right). All data are presented as mean ± SEM. *P < 0.05, ** P < 0.01, *** P < 0.001, ns, not significant. One-way ANOVA in **C–F**

To unambiguously prove the role of LRRC19 in DVF-mediated inflammation, *Lrrc19*^*−*/*−*^ mice were generated (Fig. 6A) and treated with DVF or PBS respectively (Fig. 6B). During the experiment period, no significant difference in weight loss and DAI score were observed between two groups (Fig. 6C, D). The colon length was not significantly different between DVF- and PBS-treated *Lrrc19*^*−*/*−*^ mice (Fig. 6E). Additionally, H&E staining and histological analysis showed decreased epithelial disruption and limited leukocyte infiltrations in DSS-treated *Lrrc19*^*−*/*−*^ mice of both groups, while no significant differences between groups were observed (Fig. 6F).

To further confirm the role of LRRC19 in DVF-mediated inflammation, we then treated colon organoids derived from colitis mice (*Lrrc19*^*−*/*−*^ mice) with DVF or PBS. The results showed that no significant difference was observed for the organoid per crypts ratio between 2 groups (Figure S9A). There was also no difference in the number of crypt-like domains and circumference per organoid between the two groups (Figure S9A). Besides, we found DVF challenge did not promote apoptosis in the organoids derived from colitis mice (*Lrrc19*^*−*/*−*^ mice; Figure S9B). The knockdown of LRRC19 was confirmed by immunostaining (Figure S9C). Taken together with in vitro observations, these results confirm that DVF-mediated exacerbation of colitis is dependent on activation of LRRC19 signaling.

### Blockade of DVF-LRRC19 interaction by typhaneoside alleviates the pro-inflammatory effect of DVF in mice

**Fig. 7:**
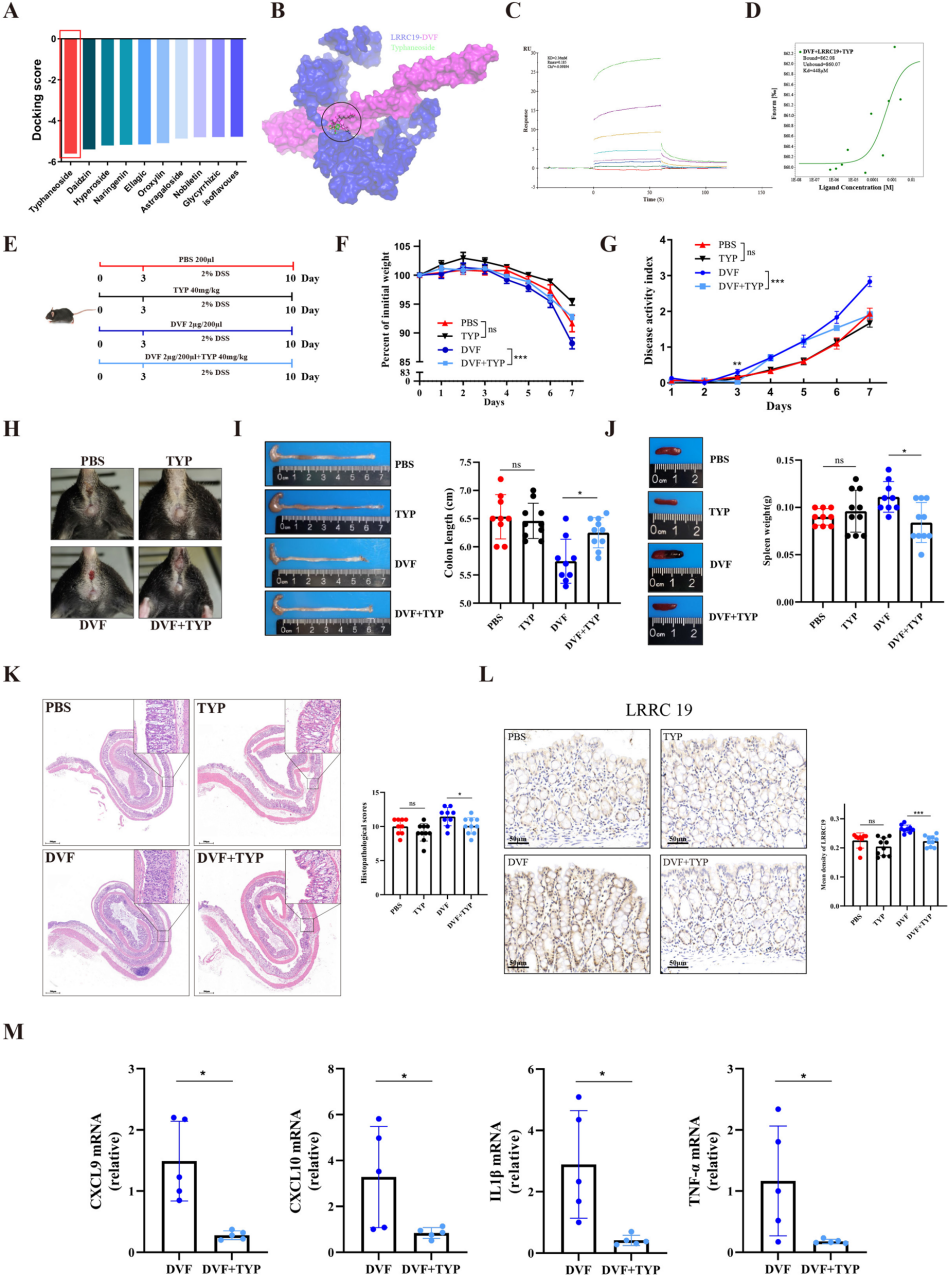
Blockade of interaction between DVF and LRRC19 by typhaneoside alleviates the pro-inflammatory effect of DVF in mice. **A** Molecular docking results of high-throughput screening based on the structure of DVF/LRRC19 complex. **B** Predicted binding modes of TYP and DVF/LRRC19 complex and its three-dimensional structure. **C** Biacore analysis of the interaction between typhaneoside and LRRC19. **D** Microscale thermophoresis result for the binding of DVF to LRRC19 in the presence of TYP. **E** The experimental design of DSS model. **F** Body weight was presented as a percentage of the initial weight. **G–J** DAI (**G**), representative images of anal bleeding (**H**), colon length (**I**), and spleen weight (**J**) are shown. **K** Representative histological images of colon tissues by H&E staining (left panel) and histopathological score (right panel). Scale bars, 500 µm. **L** IHC staining and quantitation of LRRC19 in the colonic mucosa of colitis mice. Scale bars, 50 µm. **M** The relative mRNA expression of *Cxcl9*, *Cxcl10*, *IL1β*, and *Tnf-α* in colon tissues. All data are presented as mean ± SEM. *P < 0.05, *** P < 0.001, ns, not significant. Two-tailed Student’s *t* test in **F**, **G**; one-way ANOVA in **I–L**; two-tailed Mann–Whitney *U* test in **M**

In light of the findings described previously, we found DVF-LRRC19 interaction performs critical functions in colitis pathogenesis and therefore could be served as a strategy for developing novel therapeutics. Given the inhibitor of LRRC19 has cytotoxicity, we searched potential therapeutic agent based on the DVF-LRRC19 interaction using high-throughput molecular docking virtual screening. Consequently, typhaneoside was selected according to the molecular docking scores (Fig. 7A). Molecular docking model of the typhaneoside targeting DVF-LRRC19 complex is shown in Fig. 7B. To validate the modeling results, we performed Biacore and MST assays. SPR results indicated typhaneoside could not directly bind to LRRC19 (Fig. 7C), while MST results showed the binding affinity between DVF and LRRC19 was reduced approximately 400-fold by typhaneoside (Fig. 7D). These data suggest that typhaneoside could influence the interaction between DVF and LRRC19.

Typhaneoside is a flavonoid glycoside extracted from *Typha angustifolia L*. [31]. To further demonstrate the inhibitory effect of typhaneoside on DVF-LRRC19 interaction in vivo, we designed animal experiments (Fig. 7E). Surprisingly, typhaneoside treatment alone did not drastically improve weight loss and DAI scores in colitis mice (Fig. 7F, G). Of note is that typhaneoside significantly prevented DVF-induced exacerbation of weight loss, DAI scores, and anal bleeding (Fig. 7F–H). The shortened colon length, increased spleen weight, and expanded inflammatory cell infiltration after DVF challenge were also significantly blunted by typhaneoside (Fig. 7I–K). Together, these results indicate that typhaneoside could suppress DVF-induced aggravation of colitis but not colitis alone.

Not unexpectedly, the increased expression of LRRC19 by DVF could be suppressed by Typhaneoside, suggesting a blocking effect of Typhaneoside on the combination of DVF and LRRC19 (Fig. 7L). However, typhaneoside treatment had no directly inhibitory effect on LRRC19 expression in colitis mice (Fig. 7L). In addition, the upregulated expression levels of downstream genes in colitis mice after DVF challenge, including *Cxcl9*, *Cxcl10*, *IL1β*, and *Tnf-α*, were significantly decreased by typhaneoside (Fig. 7M). Overall, we concluded that typhaneoside could block the DVF-LRRC19 interaction and thus alleviate the pro-inflammatory effect of DVF in colitis mice.

## Discussion

Increasing numbers of studies have provided evidence that gut dysbiosis is intimately related to the pathogenesis of UC [32]. Several studies have shown increased *Desulfovibrio* spp. in the feces of UC patients [5, 6, 33]. However, the underlying mechanism has not been well documented. LRRC19 belongs to the immune recognition receptors and has recently been linked to inflammatory bowel disorders [34]. In this study, we found that enrichment of *D. vulgaris* was accompanied by upregulation of LRRC19 in the mucosa of UC patients. Our data indicated the flagellin of *D. vulgaris* could interact with LRRC19 and thus accelerate colitis, suggesting a key role of *D. vulgaris* in the pathogenesis of UC.

Prior studies have noted *D. vulgaris* is increased in fecal samples of UC [4]. In line with this, we also found *D. vulgaris* was increased in feces of UC and was correlated with disease severity. *D. vulgaris* has been reported to increase gut H_2_S levels, which can inhibit butyrate metabolism in colonocytes, alter intestinal lumen pH, and thus cause intestinal inflammation [35]. However, it is unclear whether and how flagellin of *D. vulgaris* promoted the development of colitis. Emerging evidence suggests that bacterial flagellin plays a vital role in inducing dysregulated immune response in IBD [36, 37]. In this experiment, we reported DVF can exacerbate inflammation of DSS-induced colitis in mice. Such a pro-inflammatory role of DVF was also substantiated by in vitro experiments. More specifically, a recent study has also demonstrated that the flagellin of adherent-invasive *E. coli* (AIEC) are required in the AIEC-induced inflammation [25]. Hence, we proposed that DVF might have important role in promoting inflammation. Investigations of the impact of *D. vulgaris* flagellar mutant on colitis are underway in our laboratory.

It is well known that TLR5 can recognize bacterial flagellin and activate innate immune response [11]. Nevertheless, the role of TLR5 in UC was controversial. Several studies have shown that mice deficient in TLR5 were prone to developing spontaneous colitis [38, 39]. More importantly, Ivison et al. found that DSS colitis was more severe in *Tlr5*^*−*/*−*^ mice and flagellin-mediated exacerbation of colitis is independent of TLR5 [28]. Therefore, we assumed DVF-mediated inflammation was not likely to be induced by TLR5 activation. Further support for this hypothesis comes from our in vivo and in vitro experiments, in which the expression levels of TLR5 was not increased after DVF stimulation and DVF significantly worsened the severity of DSS-induced colitis in *Tlr5*^*−*/*−*^ mice. LRRC19 is a PRR mainly expressed in intestinal epithelium. Our results suggested that DVF could interact with LRRC19 using molecular docking analysis, which was further verified by SPR and MST assays. Moreover, we observed the expression levels of LRRC19 was significantly increased both in vivo and in vitro experiments after DVF challenge, this would support that DVF may directly interact with LRRC19 and thus contribute to colonic inflammation.

Previous study has demonstrated that LRRC19 can recognize lipopolysaccharide (LPS) and mediate the activation of NF-κB and MAPK pathways through inhibiting degradation of TRAF2 and increasing K63-linked ubiquitin on TRAF6 [15]. Interestingly, we uncovered that DVF treatment could mediate K63-linked ubiquitination of TARF6, but K48-linked ubiquitin did not appear in the TRAF2. This suggests a differential activation of LRRC19 downstream pathways, mainly dependent on TRAF6. It has been well established that TLRs can bind different ligands (pathogen-associated molecular patterns, PAMPs) and recruit specific adaptors to initiate the downstream signaling pathways [11]. Critically, TLR4 can recognize a wide range of PAMPs (such as LPS, viral glycoproteins, and fibronectin) and recruit different cytosolic adaptors (such as myeloid differentiation primary response 88 (MyD88) and TIR domain-containing adaptor-inducing IFN-β related adaptor molecule). Combined, our data provide a valuable mechanistic explanation that DVF could interact with LRRC19 and then recruit different cytosolic adaptors distinct from LPS.

Activation of NF-κB and MAPK pathways may lead to the production of pro-inflammatory chemokines and cytokines [40, 41]. In this study, we found the expression levels of pro-inflammatory chemokines downstream of the LRRC19 pathway were markedly elevated after DVF treatment. Subsequently, DVF-induced increased chemokines promoted the recruitment of pro-inflammatory immune cells, which may contribute to inflammatory responses in colitis [42–44]. Thus, our data demonstrated DVF can initiate the MAPK and NF-κB cascades, promote the recruitment of immune cells and the production of pro-inflammatory cytokines, and thus accelerate colitis development. On the other hand, a decreased susceptibility to colitis was observed in *Lrrc19*^*−*/*−*^ mice [13]. Our data showed siRNA-mediated knockdown of LRRC19 or *Lrrc19*^*−*/*−*^ mice dramatically abolished DVF-mediated activation of MAPK and NF-κB pathway and its associated inflammation, supporting the notion that LRRC19 is a critical signaling component in DVF-induced inflammation.

Significant advances have been made in the development of new targeted therapeutic agents for IBD [45, 46]. Developing drugs that target protein–protein interaction is becoming more widespread [47, 48]. Recently, modulation of protein–protein interaction by small molecules or TCM monomer has received substantial attention [49, 50]. Thus, based on our findings, we identified typhaneoside as the blockade of DVF-LRRC19 interaction. Typhaneoside have been considered to have anti-inflammatory and anti-oxidative stress effects [31, 51]. In the present study, although typhaneoside treatment did not significantly improve DSS colitis in mice, we found typhaneoside can prevent DVF-mediated activation of LRRC19 signaling and exacerbation of colonic inflammation. Putting these characteristics together, we propose typhaneoside as a therapeutic candidate for colitis induced by *D. vulgaris* or DVF. Further pharmacological experiments need to be conducted to explore the clinical use of typhaneoside.

## Conclusions

**Fig. 8:**
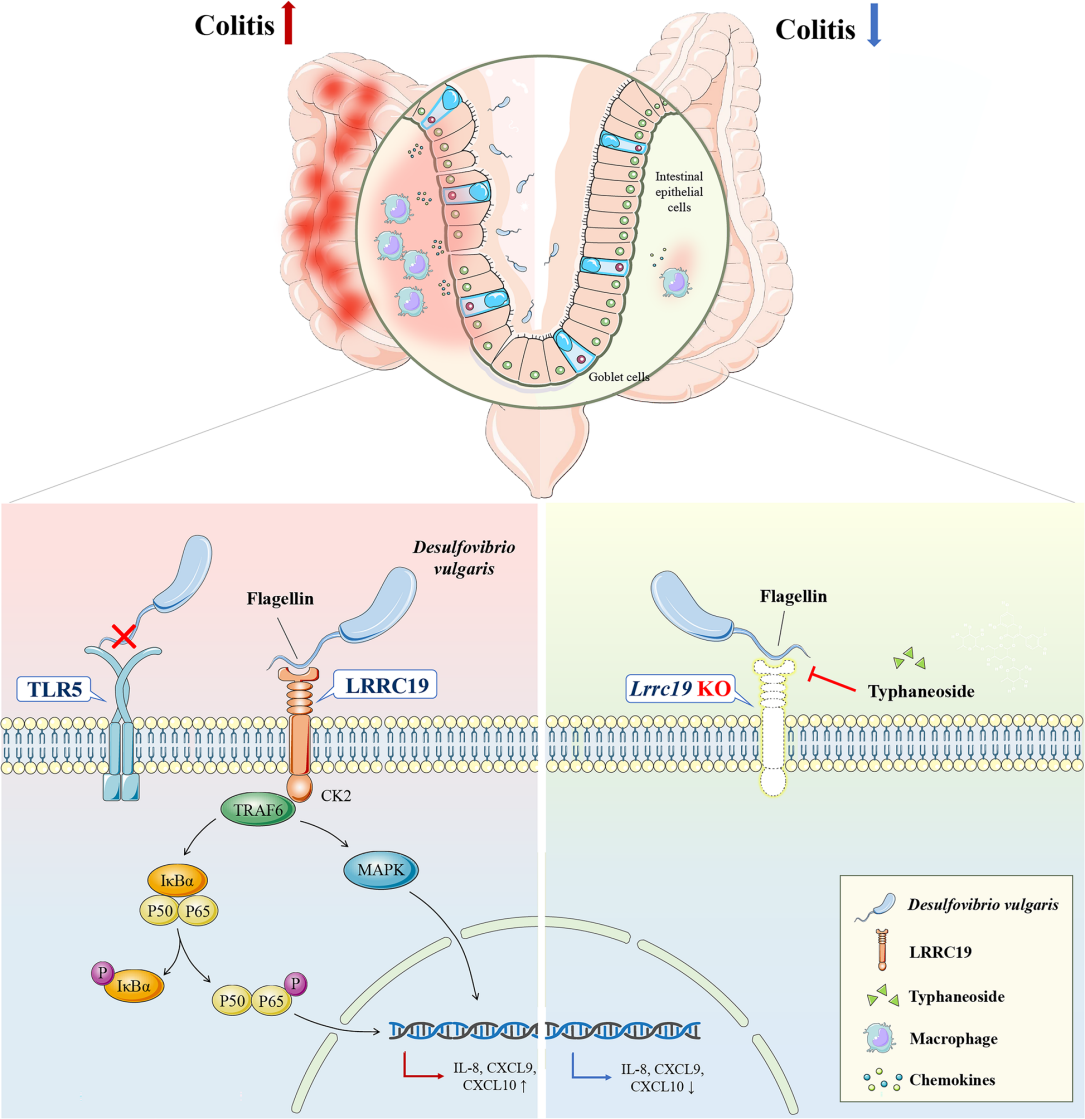
Schematic summary for the role of DVF-LRRC19 interaction in the pathogenesis of colitis

In summary, we have found *D. vulgaris* was increased in the feces of UC patients. Our data indicate DVF can combine with LRRC19, and then direct interact with TRAF6 and activate the MAPK/NF-κB pathway, which in turn promote the recruitment of immune cells and production of pro-inflammatory cytokines, and thus aggravate colonic inflammation (an overview of mechanism diagram is shown in Fig. 8). Importantly, our work also identifies typhaneoside might have potential therapeutic effects in those UC patients with high amount of *D. vulgaris*.

### Supplementary Information

**Additional file 1:** **Supplementary Figure S1. **Generation and identification of DVF. (A) The recombinant plasmids were digested by MluI and XhoI and analyzed on an agarose gel. (B) Purified DVF was eluted with imidazole and the eluates were probed on western blots.  

**Additional file 2:** **Supplementary Figure S2. ***D. vulgaris* promotes colitis in germ-free mice.(A) The experimental design of DSS model in germ-free mice. (B) Body weight was presented as a percentage of the initial weight.(C-D) DAI (C) and colon length (D) were shown. (E) Representative histological images of colon tissues by H&E staining (left panel) and histopathological score (right panel). Scale bars, 100 µm. All data are presented as mean±SEM. *P <0.05, ** P <0.01. Two-tailed Student’s t-test in (B-E). DSV: *Desulfovibrio vulgaris*. 

**Additional file 3:** **Supplementary Figure S3. **Effects of DVF on the fecal microbiota in mice without colitis. (A) Chao1 and Shannon index of alpha diversity. (B) Relative abundance of bacteria at the phylum level. (C) Principal Coordinate Analysis (PCoA) based on Weighted UniFrac distances. (D-E) Cladogram representing taxa of the two groups (D) and LDA scores was determined by Linear Discriminant Analysis Effect Size (LEfSe) analysis, the cutoff value is the absolute log10 LDA score>2.0 (E). (F) Functional prediction of fecal microbiota using PICRUSt2.

**Additional file 4:** **Supplementary Figure S4.** DVF leads to activation of inflammatory genes. (A) The relative mRNA expression of *Lrrc19*, *Cxcl9*, *Cxcl10*, *IL1β*, and *Tnf-α*in colon tissues was validated by RT-PCR. (B) Protein levels of IL1β, CXCL9, and CXCL10 in colon tissues was validated by western blotting, α-tubulin was used as loading control. (C) Protein levels of TNF-α in colon tissues was measured by ELISA. All data are presented as mean±SEM. *P <0.05, ** P <0.01. one-way ANOVA in (A-C).

**Additional file 5:** **Supplementary Figure S5.** DVF does not induce the increased expression of TLR5. (A) RNA-seq results of *Tlr5 *mRNA. (B) The relative mRNA expression of *Tlr5*in colon tissues of colitis mice was validated by RT-PCR. (C) The relative mRNA expression of *Tlr5* in Caco2 cells after treated with DVF. (D) Mouse genotypes were determined by PCR using the indicated primers to detect wild-type and mutant alleles of *Tlr5*. (E) The experimental design of DSS model in WTand *Tlr5*^−-/−-^ mice. (F) Body weight was presented as a percentage of the initial weight. (G-H) DAI (G), and colon length (H) were shown. (I) Representative histological images of colon tissues by H&E staining (left panel) and histopathological score (right panel). Scale bars, 500 µm. All data are presented as mean±SEM. *P <0.05, ** P <0.01, *** P<0.001; ns, not significant. Two-tailed Student’s t-test in (B-C), one-way ANOVA in (F-I).

**Additional file 6:** **Supplementary Figure S6.** DVF interacts with LRRC19. (A-C) Protein–protein docking of DVF and LRRC19 (A-B) and the interaction interface of amino acid in the binding site (C).

**Additional file 7:** **Supplementary Figure S7.** DVF activates LRRC19/TRAF6 signaling. (A) The relative mRNA expression levels of LRRC19 in Caco2 cells after treated with DVF. (B) Protein levels of LRRC19 in Caco2 cells after control or LRRC19 siRNA transfection with/or without DVF treatment were measured by western blotting, α-tubulin was used as loading control. (C) Immunoprecipitation and immunoblotting for the level of K48-linked ubiquitination and K63-linked ubiquitination in Caco2 cells after treated with DVF. Immunoprecipitation was performed with anti-TRAF2 or anti-TRAF6, immunoblotting was performed for the level of LRRC19, TRAF2, TRAF6, K48-linked ubiquitination with TRAF2 (left), and K63-linked ubiquitination with TRAF6 (right). GAPDH was used as loading control. IB, immunoblotting; IP, immunoprecipitation. All data are presented as mean±SEM. *P <0.05, ** P <0.01; ns, not significant. Two-tailed Student’s t-test in (A-B).

**Additional file 8:** **Supplementary Figure S8.** DVF activates the MAPK/NF-κB pathway and induces the production of pro-inflammatory chemokine and cytokines. (A) Protein levels of P-p38, P-ERK, P-NF-κB p65, and P-IκBα in Caco2 cells after control or LRRC19 siRNA transfection with/or without DVF treatment were measured by western blotting, α-tubulin was used as loading control. (B) The relative mRNA expression of *Cxcl9*, *Cxcl10*, *IL1β*, and *IL8* in Caco2 cells after treated with DVF. (C) The relative mRNA expression of *Cxcl9*, *Cxcl10*, *IL1β*, and *IL8* in NCM460 cells after treated with DVF. (D) The relative mRNA expression of *Lrrc19* in Caco2 cells after LRRC19 siRNA transfection. All data are presented as mean±SEM. *P <0.05,** P <0.01; ns, not significant. Two-tailed Student’s t-test in (A-D). 

**Additional file 9:** **Supplementary Figure S9.** DVF promotes apoptosis in the organoids derived from colitis mice and increases the expression of LRRC19. (A) The size and number of organoids derived from colitis mice (WT mice and LRRC19 knockout mice) with/or without DVF treatment. (B) *The proportion of apoptotic cells in *organoids* was assessed by Annexin V-FITC staining.* (C) The expression of LRRC19*in *organoids after treated with DVF was assessed by immunostaining (red; scale bars: 10 µm). All data are presented as mean±SEM. *P <0.05, ** P <0.01, ns, not significant. one-way ANOVA in (A-B).

**Additional file 10:** **Supplementary Figure S10**. LRRC19 expression is upregulated in colonic tissues from UC patients. (A) TLR5 expression was not upregulated in tissue samples from UC patients by NCBI GEO database (GSE42911 and GSE105074). (B) LRRC19 expression was upregulated in tissue samples from UC patients by NCBI GEO database (GSE42911 and GSE105074). (C) IHC staining and quantitation of LRRC19 in the colonic mucosa of UC patients. Scale bars, 100 µm. All data are presented as mean±SEM. *P<0.05, ns, not significant. Two-tailed Student’s t-test in (A-C).

**Additional file 11:** **Supplementary Figure S11**. DVF promotes the recruitment of inflammatory immune cells in mice. (A) The percentages of Ly6C^+^MHCII^+^cells and CD103^+^CD11b^+^ DCs in CLP of DVF or PBS treated colitis mice. (B-C) The percentages of CD103^+^CD11b^+^ DCs in MLN (B) and PPs (C) of DVF or PBS treated mice with colitis. All data are presented as mean±SEM. *P <0.05, ** P <0.01. Two-tailed Student’s t-test in (A-C).

**Additional file 12:** **Supplementary Figure S12.** FCM gating strategies.

**Additional file 13:** **Supplementary Table 1.** The Characteristics of UC patients and health controls in this study. **Supplementary Table 2**. Clinical Characteristics of the Included UC Patients. **Supplementary Table 3.** The primers used in this study. **Supplementary Table 4.** The antibodies used in flow cytometry. **Supplementary Table 5.** Docking scores among different flagellin and flagellin receptor.

## Data Availability

All data are available from the corresponding author upon reasonable request. The RNA-seq data (PRJNA757186) and 16S rRNA data (PRJNA756633) are deposited with the NCBI and are available for download. The sequence of DVF is available on the NCBI (AE017285.1).

## Abbreviations

UC: Ulcerative colitis
*D. vulgaris* (DSV): *Desulfovibrio vulgaris*
DVF: *Desulfovibrio vulgaris* Flagellin
LRRC19: Leucine-rich repeat containing 19
TLR5: Toll-like receptors 5
IBD: Inflammatory bowel disease
HFD: High-fat diet
SRB: Sulfate-reducing bacteria
H_2_S: Hydrogen sulfide
PRRs: Pattern recognition receptors
TLRs: Toll-like receptors
MAPK: Mitogen-activated protein kinases
NF-κB: Nuclear factor kappa-light-chain-enhancer of activated B cells
DSS: Dextran sulfate sodium
ATCC: American type culture collection
*E. coli*: *Escherichia coli*
LB: Luria broth
DAI: Disease activity index
FC: Fecal levels of calprotectin
CRP: C-reactive protein
ESR: Erythrocyte sedimentation rate
TCM: Traditional Chinese medicine
ANOVA: Analysis of variance
WT: Wild-type
TYP: Typhaneoside
IHC: Immunohistochemistry
siRNA: Small interfering RNA
HCs: Healthy controls
CLP: Colon lamina propria
LDA: Linear discriminant analysis
FMO: Fluorescence minus one
PPs: Peyer patches
DCs: Dendritic cells
MLN: Mesenteric lymph nodes
KEGG: Kyoto encyclopedia of genes and genomes
GO: Gene ontology
DEGs: Differential expressed genes
MST: Microscale thermophoresis
SPR: Surface plasmon resonance
GO: Gene ontology
DEGs: Differential expressed genes
TRAF2: Tumor necrosis factor receptor associated factor 2
TRAF6: Tumor necrosis factor receptor associated factor 6
GF: Germ-free
