# Supplementary figures and images for: Desulfovibrio vulgaris interacts with novel gut epithelial immune receptor LRRC19 and exacerbates colitis
Source: Microbiome. 2024 Jan 3;12:4. doi: 10.1186/s40168-023-01722-8 (PMC10763354; doi:10.1186/s40168-023-01722-8)

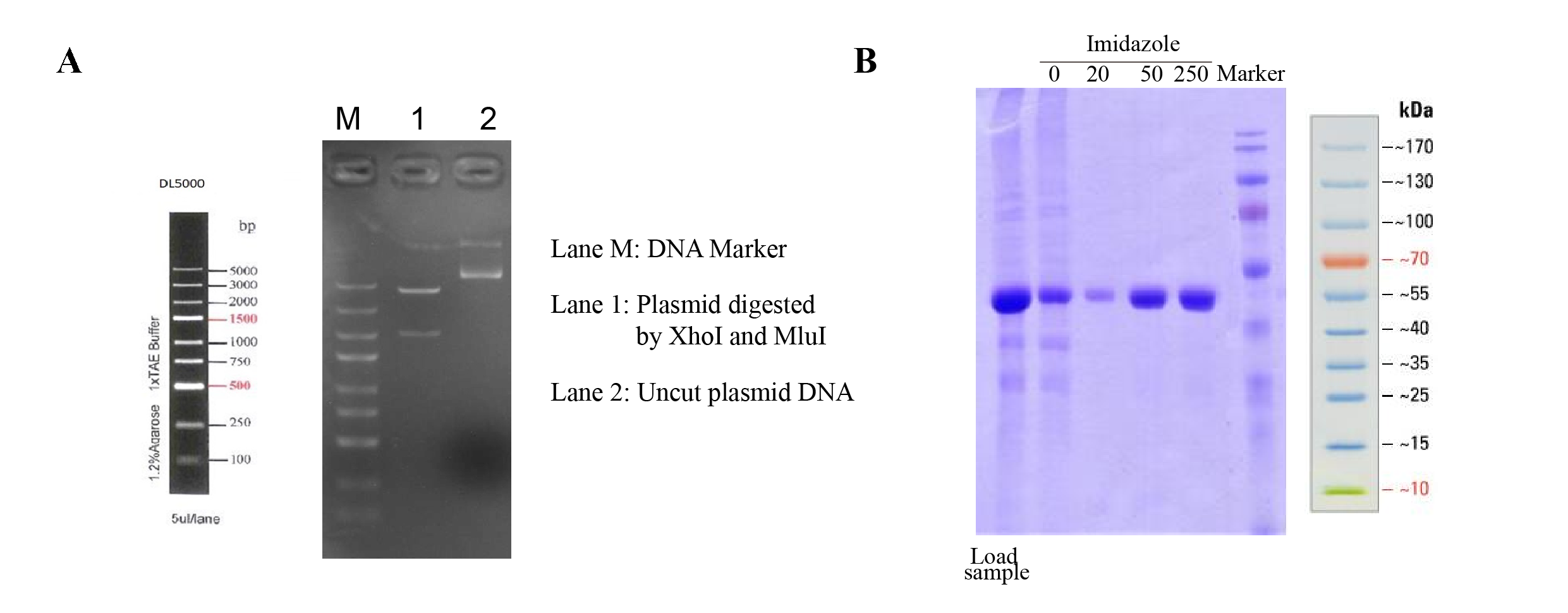

Supplement: Supplementary file 2 — Additional file 1: Supplementary Figure S1. Generation and identification of DVF. (A) The recombinant plasmids were digested by MluI and XhoI and analyzed on an agarose gel. (B) Purified DVF was eluted with imidazole and the eluates were probed on western blots. [file 40168_2023_1722_MOESM1_ESM.tif]

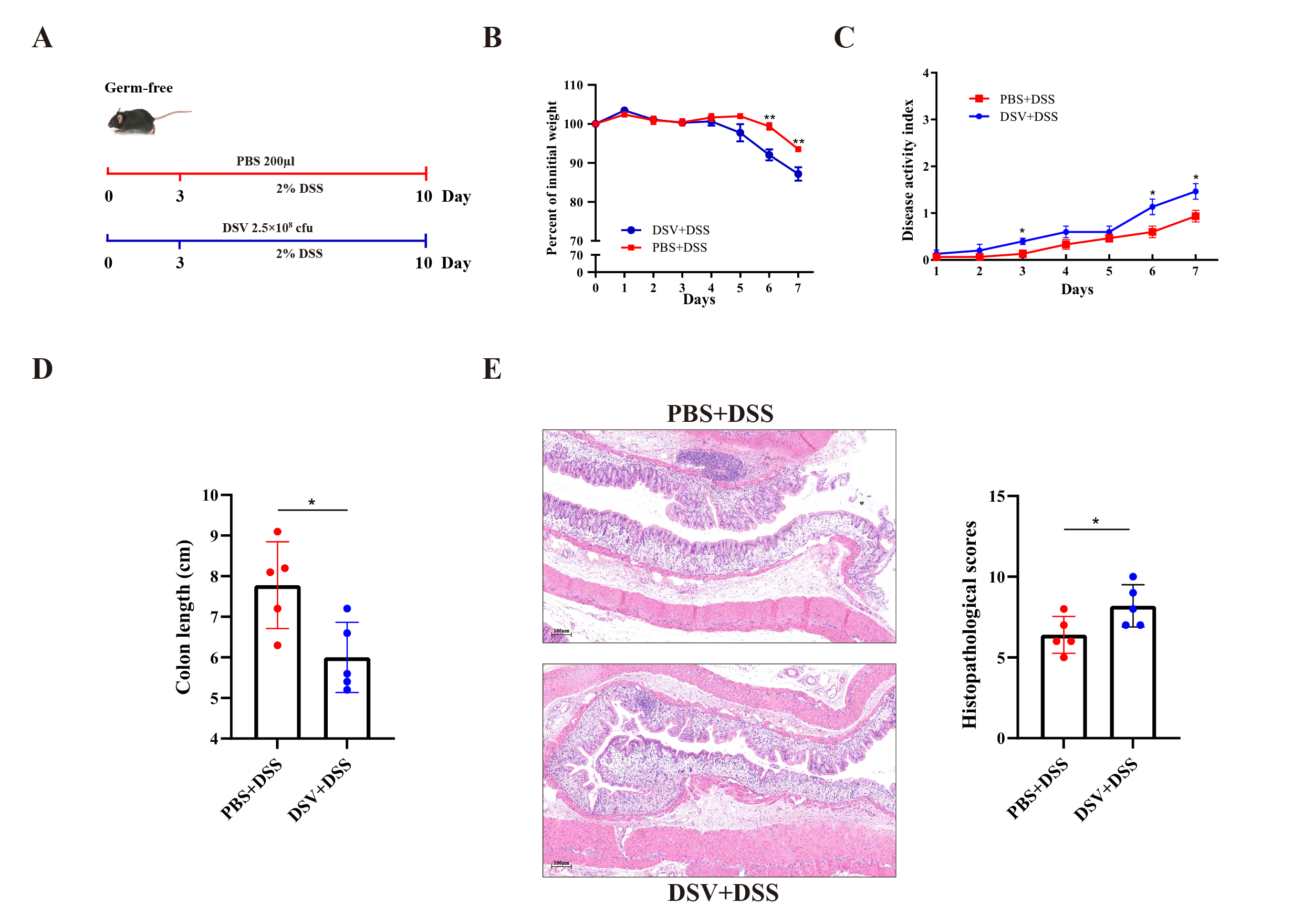

Supplement: Supplementary file 3 — Additional file 2: Supplementary Figure S2. D. vulgaris promotes colitis in germ-free mice.(A) The experimental design of DSS model in germ-free mice. (B) Body weight was presented as a percentage of the initial weight.(C-D) DAI (C) and colon length (D) were shown. (E) Representative histological images of colon tissues by H&E staining (left panel) and histopathological score (right panel). Scale bars, 100 µm. All data are presented as mean±SEM. *P <0.05, ** P <0.01. Two-tailed Student’s t-test in (B-E). DSV: Desulfovibrio vulgaris. [file 40168_2023_1722_MOESM2_ESM.tif]

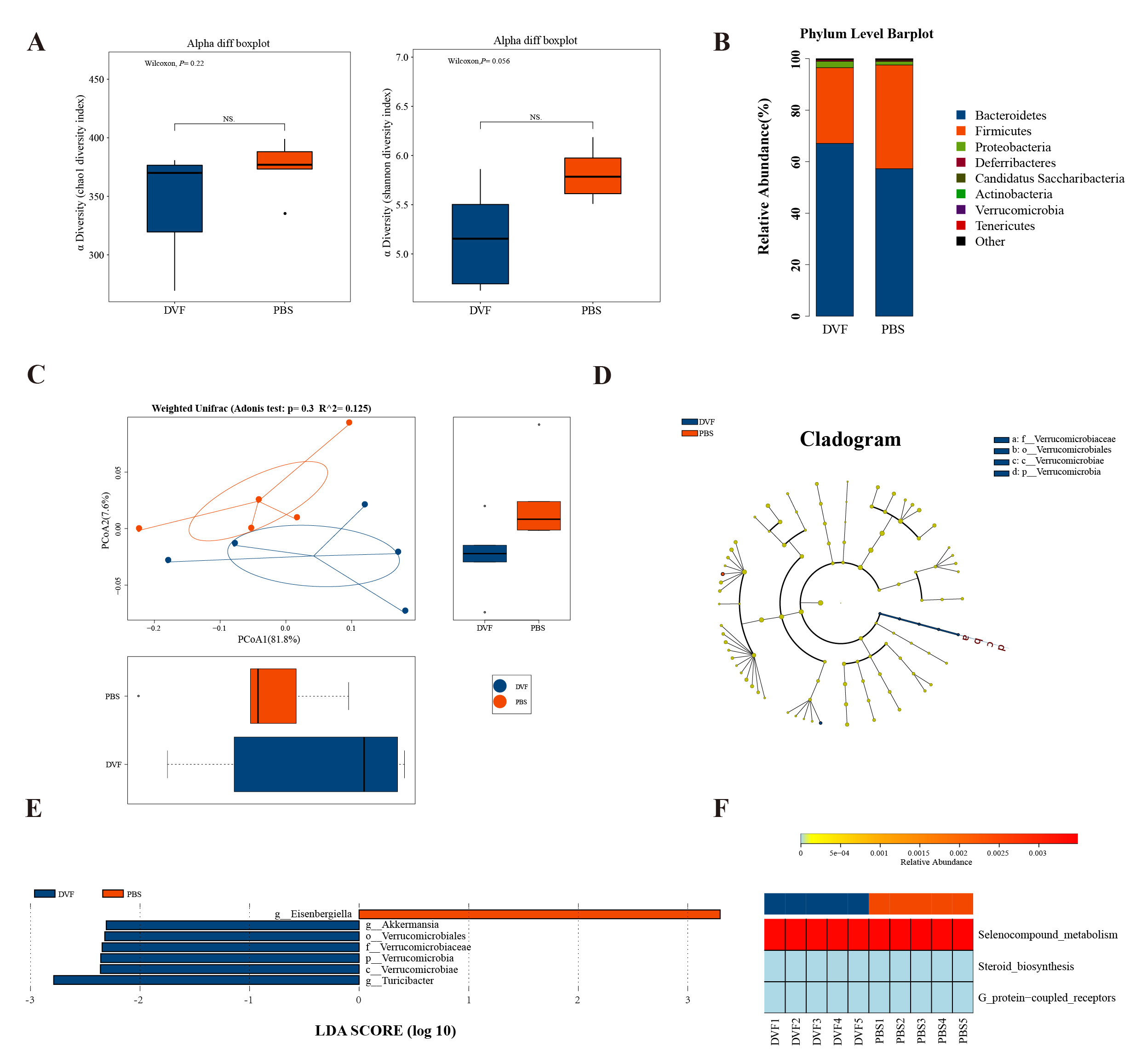

Supplement: Supplementary file 4 — Additional file 3: Supplementary Figure S3. Effects of DVF on the fecal microbiota in mice without colitis. (A) Chao1 and Shannon index of alpha diversity. (B) Relative abundance of bacteria at the phylum level. (C) Principal Coordinate Analysis (PCoA) based on Weighted UniFrac distances. (D-E) Cladogram representing taxa of the two groups (D) and LDA scores was determined by Linear Discriminant Analysis Effect Size (LEfSe) analysis, the cutoff value is the absolute log10 LDA score>2.0 (E). (F) Functional prediction of fecal microbiota using PICRUSt2. [file 40168_2023_1722_MOESM3_ESM.tif]

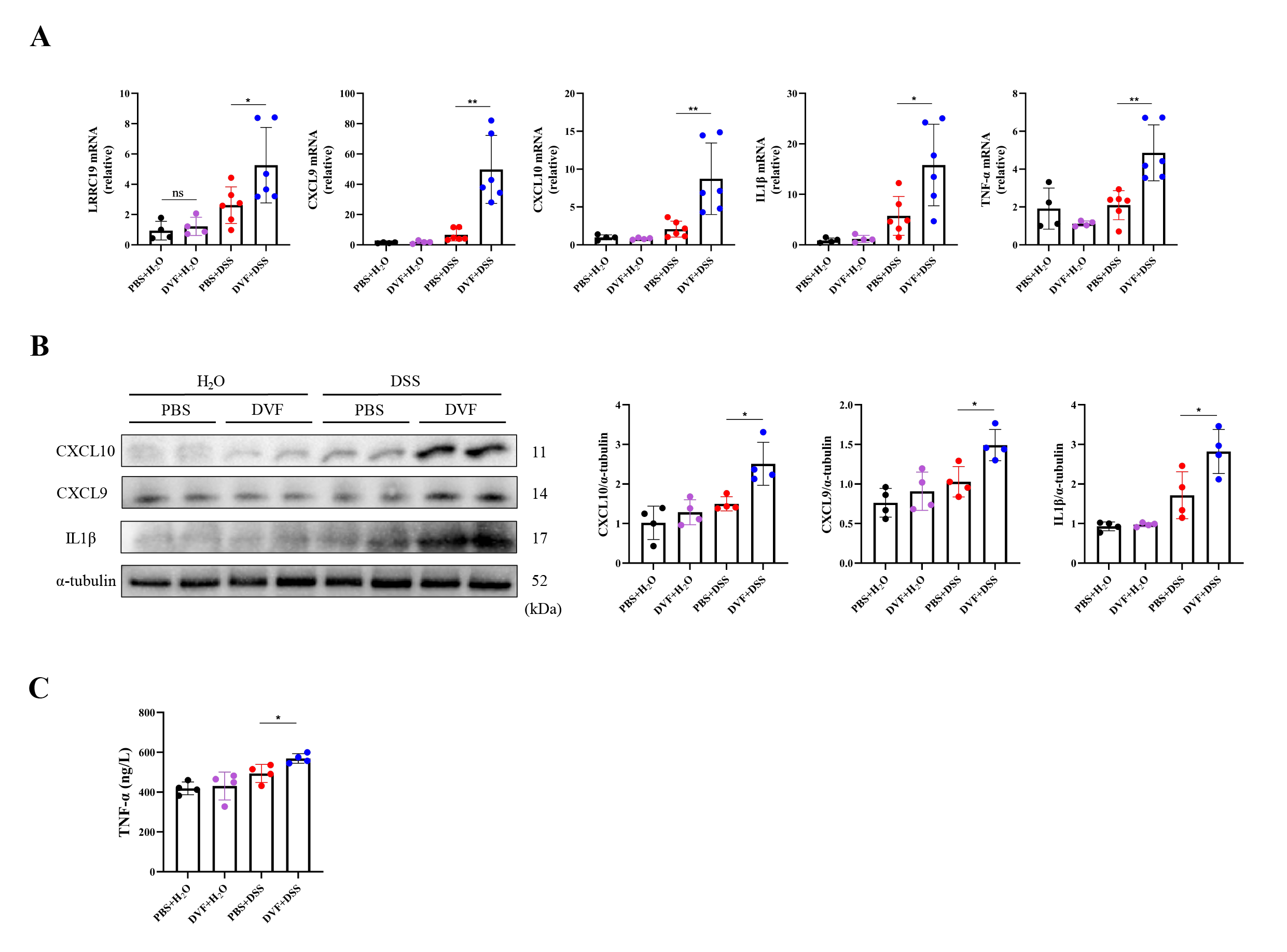

Supplement: Supplementary file 5 — Additional file 4: Supplementary Figure S4. DVF leads to activation of inflammatory genes. (A) The relative mRNA expression of Lrrc19, Cxcl9, Cxcl10, IL1β, and Tnf-αin colon tissues was validated by RT-PCR. (B) Protein levels of IL1β, CXCL9, and CXCL10 in colon tissues was validated by western blotting, α-tubulin was used as loading control. (C) Protein levels of TNF-α in colon tissues was measured by ELISA. All data are presented as mean±SEM. *P <0.05, ** P <0.01. one-way ANOVA in (A-C). [file 40168_2023_1722_MOESM4_ESM.tif]

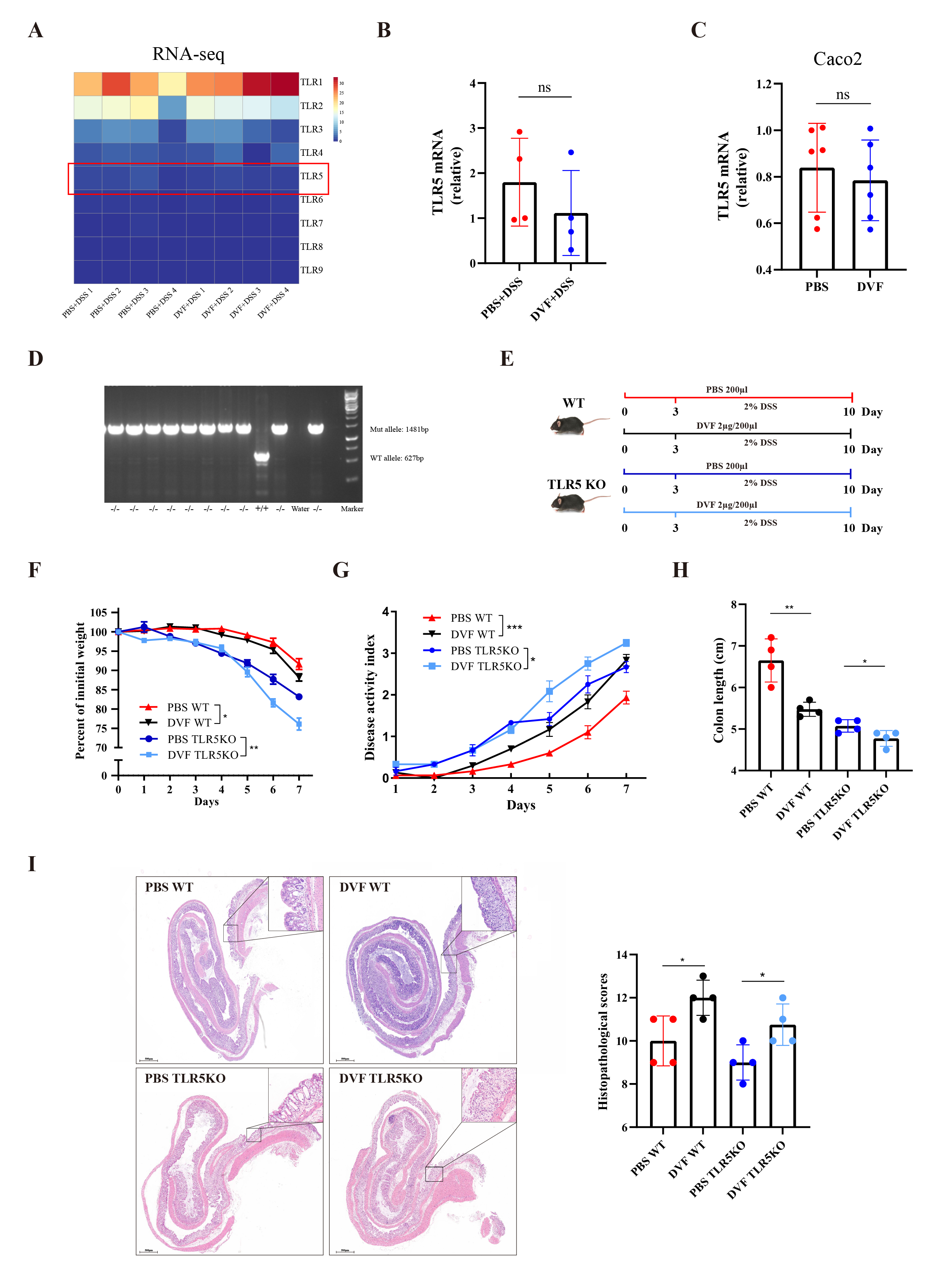

Supplement: Supplementary file 6 — Additional file 5: Supplementary Figure S5. DVF does not induce the increased expression of TLR5. (A) RNA-seq results of Tlr5 mRNA. (B) The relative mRNA expression of Tlr5in colon tissues of colitis mice was validated by RT-PCR. (C) The relative mRNA expression of Tlr5 in Caco2 cells after treated with DVF. (D) Mouse genotypes were determined by PCR using the indicated primers to detect wild-type and mutant alleles of Tlr5. (E) The experimental design of DSS model in WTand Tlr5−-/−- mice. (F) Body weight was presented as a percentage of the initial weight. (G-H) DAI (G), and colon length (H) were shown. (I) Representative histological images of colon tissues by H&E staining (left panel) and histopathological score (right panel). Scale bars, 500 µm. All data are presented as mean±SEM. *P <0.05, ** P <0.01, *** P<0.001; ns, not significant. Two-tailed Student’s t-test in (B-C), one-way ANOVA in (F-I). [file 40168_2023_1722_MOESM5_ESM.tif]

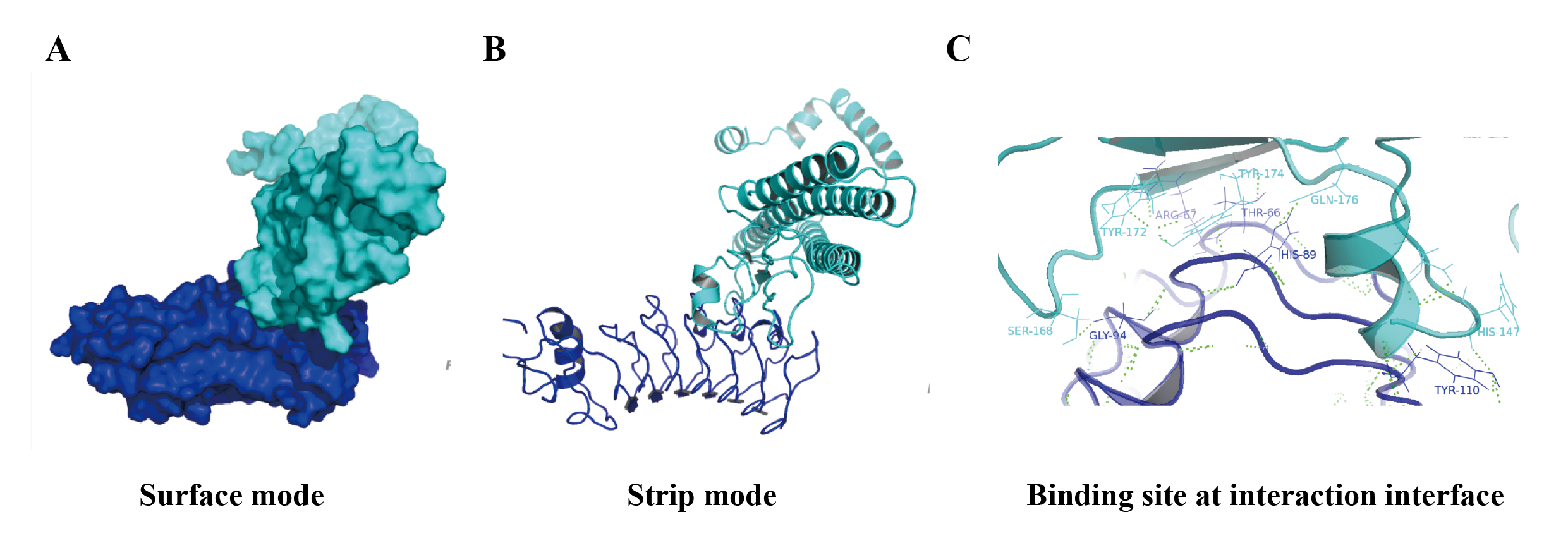

Supplement: Supplementary file 7 — Additional file 6: Supplementary Figure S6. DVF interacts with LRRC19. (A-C) Protein–protein docking of DVF and LRRC19 (A-B) and the interaction interface of amino acid in the binding site (C). [file 40168_2023_1722_MOESM6_ESM.tif]

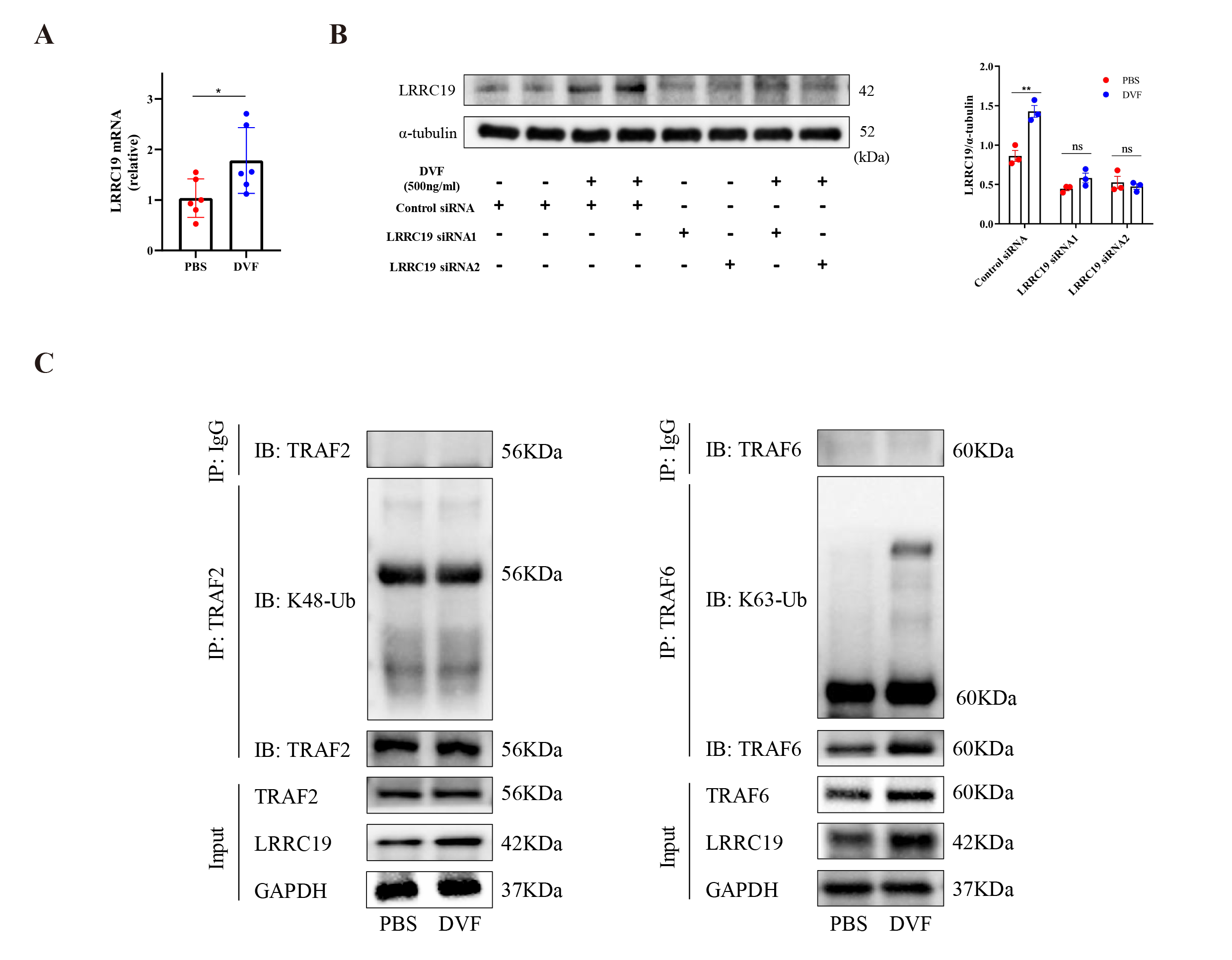

Supplement: Supplementary file 8 — Additional file 7: Supplementary Figure S7. DVF activates LRRC19/TRAF6 signaling. (A) The relative mRNA expression levels of LRRC19 in Caco2 cells after treated with DVF. (B) Protein levels of LRRC19 in Caco2 cells after control or LRRC19 siRNA transfection with/or without DVF treatment were measured by western blotting, α-tubulin was used as loading control. (C) Immunoprecipitation and immunoblotting for the level of K48-linked ubiquitination and K63-linked ubiquitination in Caco2 cells after treated with DVF. Immunoprecipitation was performed with anti-TRAF2 or anti-TRAF6, immunoblotting was performed for the level of LRRC19, TRAF2, TRAF6, K48-linked ubiquitination with TRAF2 (left), and K63-linked ubiquitination with TRAF6 (right). GAPDH was used as loading control. IB, immunoblotting; IP, immunoprecipitation. All data are presented as mean±SEM. *P <0.05, ** P <0.01; ns, not significant. Two-tailed Student’s t-test in (A-B). [file 40168_2023_1722_MOESM7_ESM.tif]

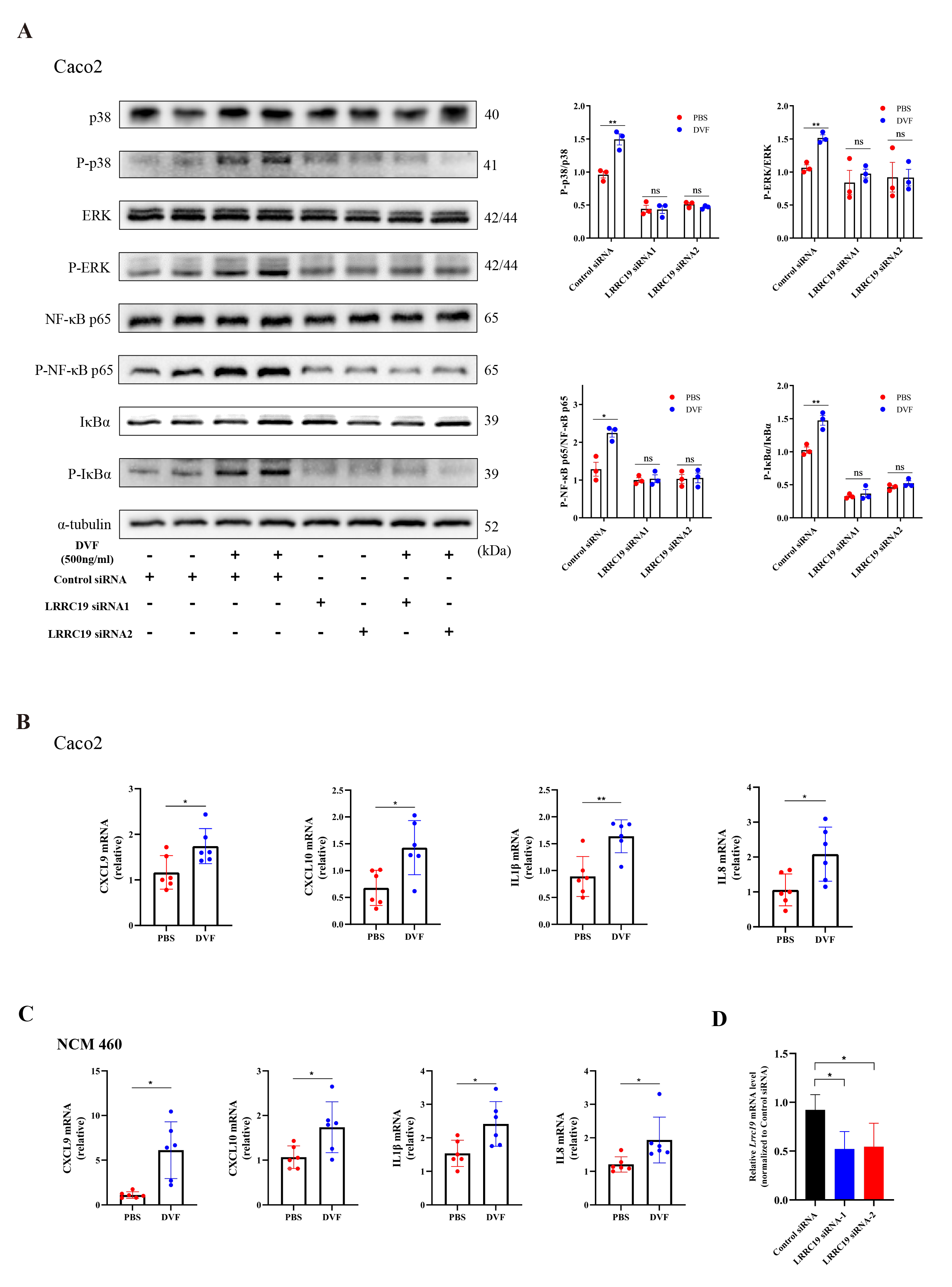

Supplement: Supplementary file 9 — Additional file 8: Supplementary Figure S8. DVF activates the MAPK/NF-κB pathway and induces the production of pro-inflammatory chemokine and cytokines. (A) Protein levels of P-p38, P-ERK, P-NF-κB p65, and P-IκBα in Caco2 cells after control or LRRC19 siRNA transfection with/or without DVF treatment were measured by western blotting, α-tubulin was used as loading control. (B) The relative mRNA expression of Cxcl9, Cxcl10, IL1β, and IL8 in Caco2 cells after treated with DVF. (C) The relative mRNA expression of Cxcl9, Cxcl10, IL1β, and IL8 in NCM460 cells after treated with DVF. (D) The relative mRNA expression of Lrrc19 in Caco2 cells after LRRC19 siRNA transfection. All data are presented as mean±SEM. *P <0.05,** P <0.01; ns, not significant. Two-tailed Student’s t-test in (A-D). [file 40168_2023_1722_MOESM8_ESM.tif]

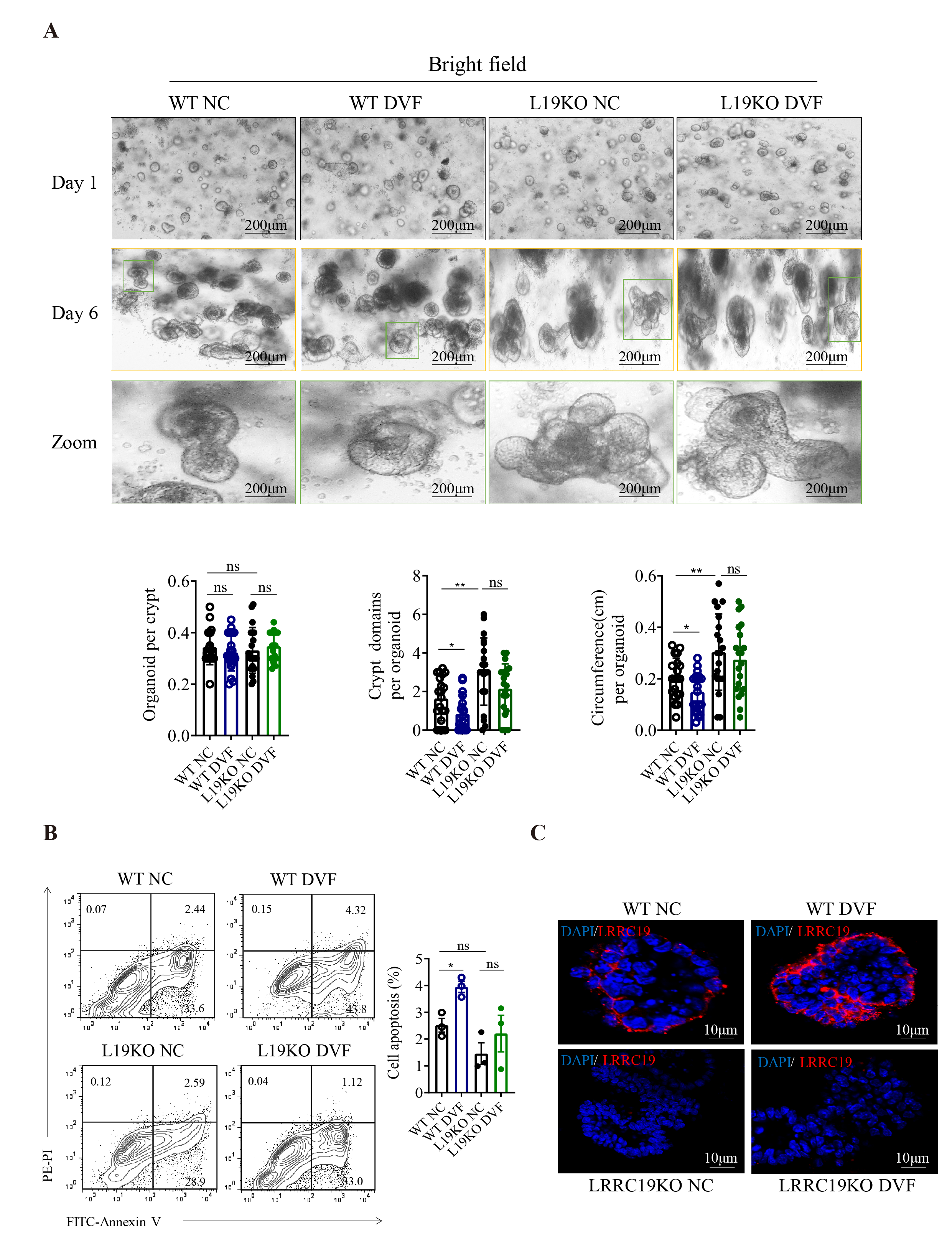

Supplement: Supplementary file 10 — Additional file 9: Supplementary Figure S9. DVF promotes apoptosis in the organoids derived from colitis mice and increases the expression of LRRC19. (A) The size and number of organoids derived from colitis mice (WT mice and LRRC19 knockout mice) with/or without DVF treatment. (B) The proportion of apoptotic cells in organoids was assessed by Annexin V-FITC staining. (C) The expression of LRRC19in organoids after treated with DVF was assessed by immunostaining (red; scale bars: 10 µm). All data are presented as mean±SEM. *P <0.05, ** P <0.01, ns, not significant. one-way ANOVA in (A-B). [file 40168_2023_1722_MOESM9_ESM.tif]

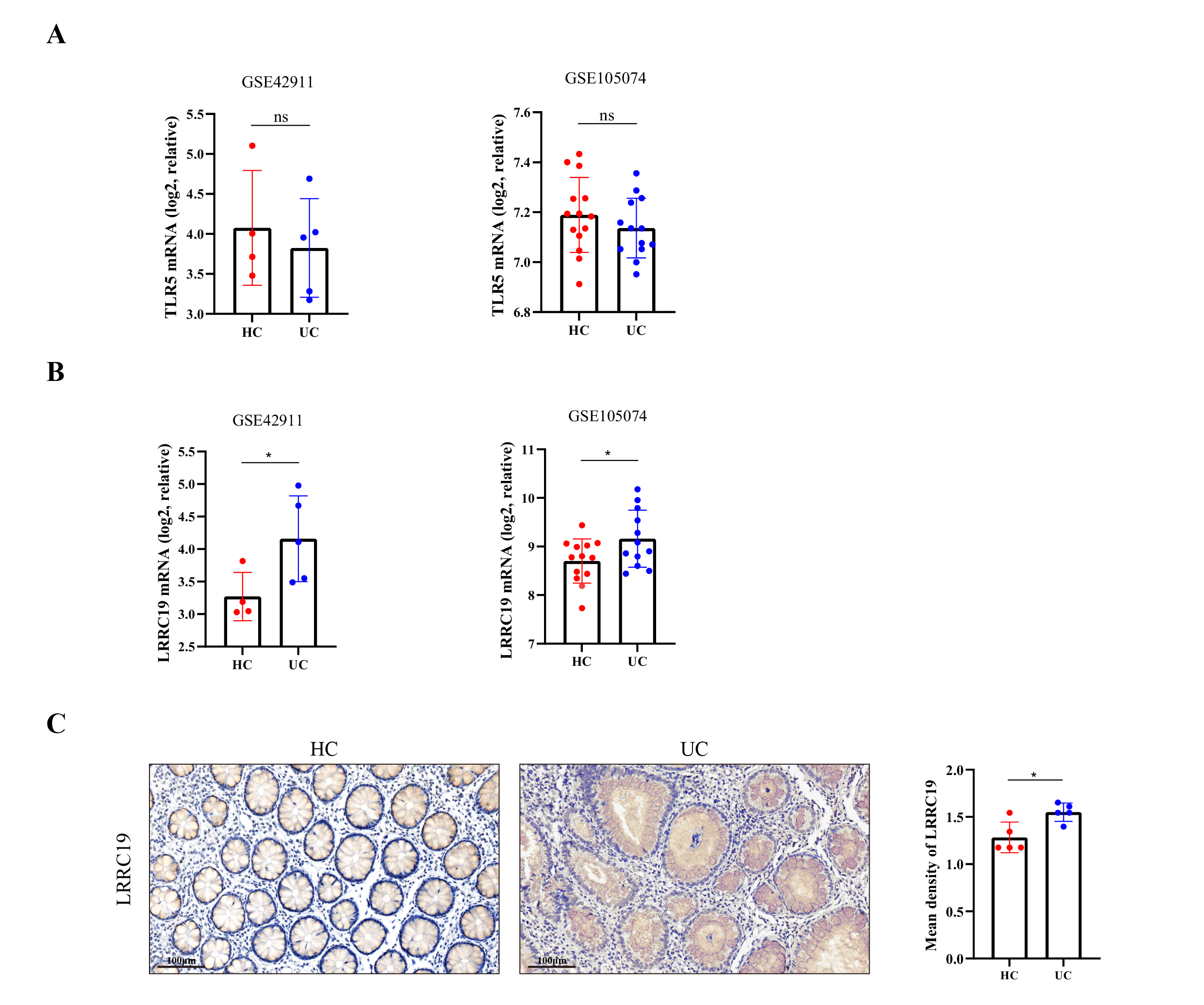

Supplement: Supplementary file 11 — Additional file 10: Supplementary Figure S10. LRRC19 expression is upregulated in colonic tissues from UC patients. (A) TLR5 expression was not upregulated in tissue samples from UC patients by NCBI GEO database (GSE42911 and GSE105074). (B) LRRC19 expression was upregulated in tissue samples from UC patients by NCBI GEO database (GSE42911 and GSE105074). (C) IHC staining and quantitation of LRRC19 in the colonic mucosa of UC patients. Scale bars, 100 µm. All data are presented as mean±SEM. *P<0.05, ns, not significant. Two-tailed Student’s t-test in (A-C). [file 40168_2023_1722_MOESM10_ESM.tif]

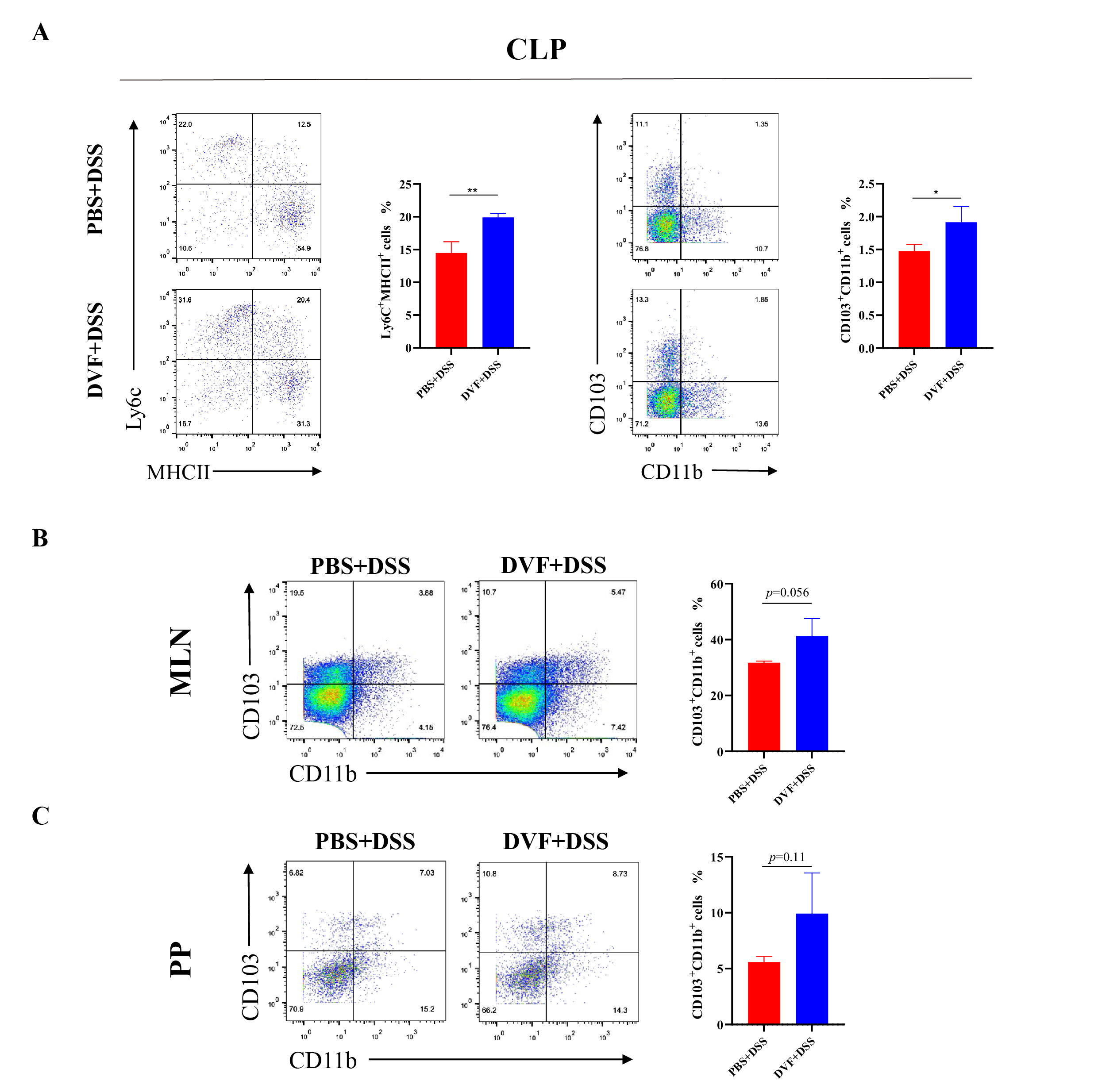

Supplement: Supplementary file 12 — Additional file 11: Supplementary Figure S11. DVF promotes the recruitment of inflammatory immune cells in mice. (A) The percentages of Ly6C+MHCII+cells and CD103+CD11b+ DCs in CLP of DVF or PBS treated colitis mice. (B-C) The percentages of CD103+CD11b+ DCs in MLN (B) and PPs (C) of DVF or PBS treated mice with colitis. All data are presented as mean±SEM. *P <0.05, ** P <0.01. Two-tailed Student’s t-test in (A-C). [file 40168_2023_1722_MOESM11_ESM.tif]

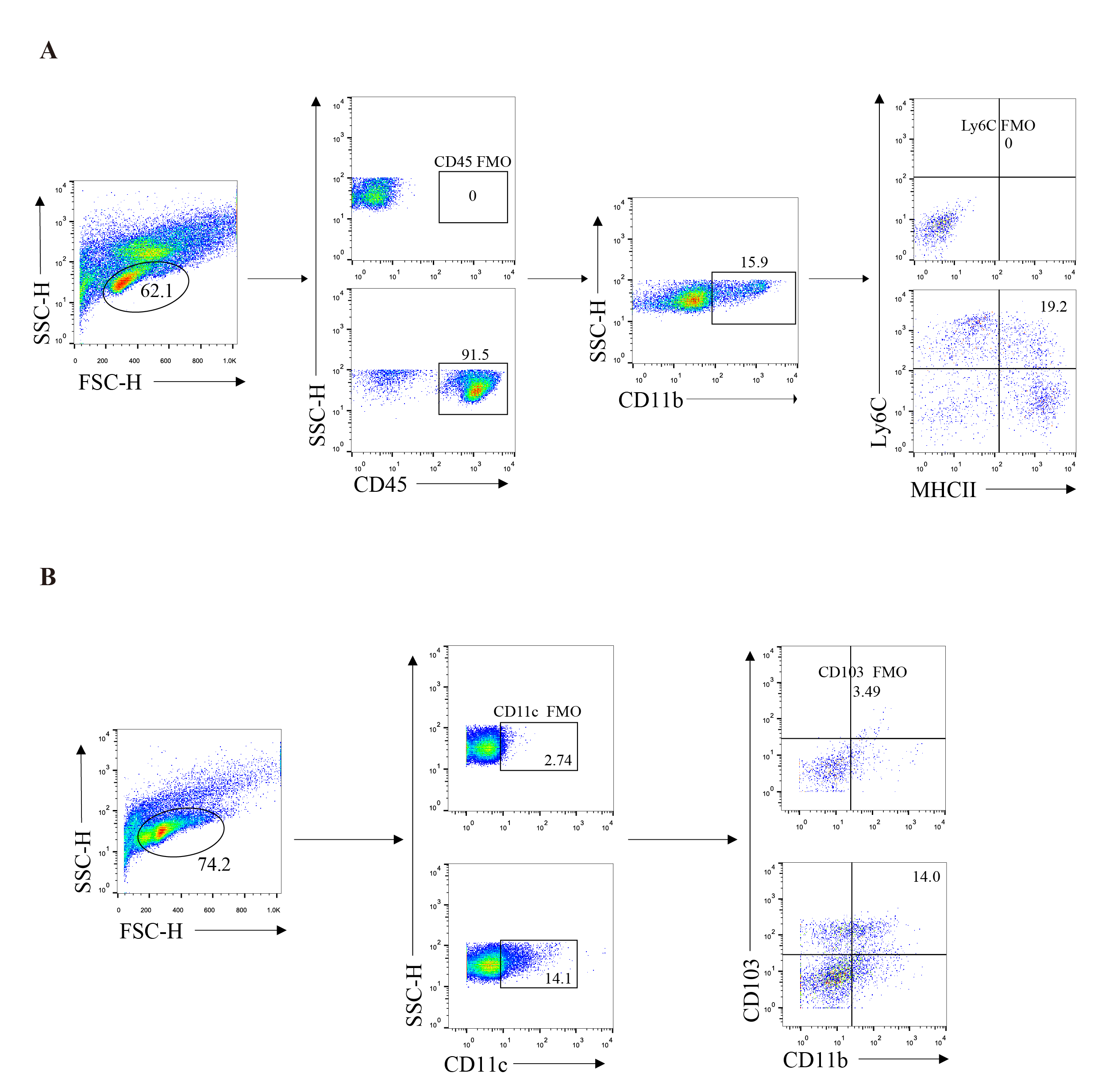

Supplement: Supplementary file 13 — Additional file 12: Supplementary Figure S12. FCM gating strategies. [file 40168_2023_1722_MOESM12_ESM.tif]
