# Supplementary material for: Desulfovibrio vulgaris interacts with novel gut epithelial immune receptor LRRC19 and exacerbates colitis
Source: Microbiome. 2024 Jan 3;12:4. doi: 10.1186/s40168-023-01722-8 (PMC10763354; doi:10.1186/s40168-023-01722-8)
Supplement: Supplementary file 14 — Additional file 13: Supplementary Table 1. The Characteristics of UC patients and health controls in this study. Supplementary Table 2. Clinical Characteristics of the Included UC Patients. Supplementary Table 3. The primers used in this study. Supplementary Table 4. The antibodies used in flow cytometry. Supplementary Table 5. Docking scores among different flagellin and flagellin receptor. [file 40168_2023_1722_MOESM13_ESM.docx]

**Supplementary Table 1.** The Characteristics of UC patients and health controls in this study

|  | UC | Health controls |
| --- | --- | --- |
| Number | 37 | 22 |
| Sex, n (%) |  |  |
| Male | 21(56.8) | 13(59.1) |
| Female | 16(43.2) | 9(40.9) |
| Age, y, mean ± SD | 45.95±13.95 (21-72) | 42.59±13.90 (23-65) |
| Disease duration (months) | 44.11±33.63 (2-120) | _ |
| Age at diagnosis (year) | 33.89±11.62 (16-59) | _ |

**Supplementary Table 2.** Clinical Characteristics of the Included UC Patients

|  | Mayo clinical score | FC (ug/g) | ESR (mm/h) | CRP (mg/dl) |
| --- | --- | --- | --- | --- |
| UC 1 | 11 | 605.5 | 34 | 0.1 |
| UC 2 | 12 | 719 | 54 | 1.66 |
| UC 3 | 4 | 234.2 | 28 | 1.12 |
| UC 4 | 12 | 818.3 | 50 | 0.19 |
| UC 5 | 11 | 599.4 | 40 | 2.82 |
| UC 6 | 8 | 178.9 | 32 | 0.94 |
| UC 7 | 12 | 657.6 | 35 | 2.14 |
| UC 8 | 12 | 93 | 49 | 0.15 |
| UC 9 | 4 | 231.5 | 9 | 2.48 |
| UC 10 | 12 | 428 | 13 | 0.1 |
| UC 11 | 6 | _ | 16 | 6.2 |
| UC 12 | 10 | 583.4 | 49 | 3.25 |
| UC 13 | 8 | 653.1 | 45 | 0.41 |
| UC 14 | 4 | 33 | 5 | 0.1 |
| UC 15 | 5 | 34.7 | 7 | 0.84 |
| UC 16 | 9 | _ | 30 | 0.74 |
| UC 17 | 7 | 823 | 18 | 0.1 |
| UC 18 | 4 | 191.9 | 54 | 4.18 |
| UC 19 | 4 | 277 | _ | 3.46 |
| UC 20 | 9 | 364 | 21 | 11.4 |
| UC 21 | 5 | 417.5 | 29 | 1.09 |
| UC 22 | 7 | 524 | _ | 0.17 |
| UC 23 | 9 | _ | _ | 0.19 |
| UC 24 | 5 | 163 | 3 | 0.31 |
| UC 25 | 6 | 514.6 | 15 | 0.45 |
| UC 26 | 4 | 44.6 | 9 | 0.47 |
| UC 27 | 11 | 812.4 | 15 | _ |
| UC 28 | 5 | 753.2 | 7 | 1.11 |
| UC 29 | 5 | 682.6 | 32 | _ |
| UC 30 | 9 | 164.9 | 13 | 0.27 |
| UC 31 | 5 | 560.3 | 32 | 1.05 |
| UC 32 | 8 | 572.2 | _ | 0.57 |
| UC 33 | 7 | _ | 27 | 0.12 |
| UC 34 | 9 | 543 | _ | _ |
| UC 35 | 7 | 394 | 5 | 0.17 |
| UC 36 | 6 | 125.8 | 35 | 0.56 |
| UC 37 | 7 | 156 | _ | _ |

**Supplementary Table 3**. The primers used in this study

|  |  | Primer sequences (5’-3’) |
| --- | --- | --- |
| Primers for Real-time PCR | Human GAPDH | Forward primer: ACATCGCTCAGACACCATG  Reverse primer: TGTAGTTGAGGTCAATGAAGGG |
|  | Human LRRC19 | Forward primer: ATTTTCAGCCCATCAGCAATTC  Reverse primer: AAGTGAAGTCGTCAGTACAGTG |
|  | Human CXCL9 | Forward primer: GGACTATCCACCTACAATCCTTG  Reverse primer: TTTTAATCAGTTCCTTCACATCTGC |
|  | Human CXCL10 | Forward primer: CCTTATCTTTCTGACTCTAAGTGGC  Reverse primer: ACGTGGACAAAATTGGCTTG |
|  | Human  IL1β | Forward primer: ATGCACCTGTACGATCACTG  Reverse primer: ACAAAGGACATGGAGAACACC |
|  | Human  IL8 | Forward primer: AGCCTTCCTGATTTCTGCAG  Reverse primer: GTCCACTCTCAATCACTCTCAG |
|  | Human  TLR5 | Forward primer: GCTAGGACAACGAGGATCATG  Reverse primer: GAGGTTGCAGAAACGATAAAAGG |
|  | Murine GAPDH | Forward primer: GGAGAAACCTGCCAAGTATG  Reverse primer: TGGGAGTTGCTGTTGAAGTC |
|  | Murine LRRC19 | Forward primer: CCTGTGGTCTACTTGAGTTGC  Reverse primer: TGTAGAGTGGCATTCAGTTGTG |
|  | Murine  CXCL9 | Forward primer: AGTCCGCTGTTCTTTTCCTC  Reverse primer: TGAGGTCTTTGAGGGATTTGTAG |
|  | Murine CXCL10 | Forward primer: CCAAGTGCTGCCGTCATTTTC  Reverse primer: GGCTCGCAGGGATGATTTCAA |
|  | Murine  IL1β | Forward primer: TCCTGTGTAATGAAAGACGGC  Reverse primer: ACTCCACTTTGCTCTTGACTTC |
|  | Murine  TNF-α | Forward primer: CTTCTGTCTACTGAACTTCGGG  Reverse primer: CAGGCTTGTCACTCGAATTTTG |
|  | Murine  TLR5 | Forward primer: TGGGGACCCAGTATGCTAACT  Reverse primer: CCACAGGAAAACAGCCGAAGT |
| Primers for *Desulfovibrio vulgaris* quantification. | *Desulfovibrio vulgaris* | Forward primer: GGCATCTGTAGACCTCCTTGTAGTC  Reverse primer: TGTCGATCGTAGGTAGCAAATGGCG |
|  | 16S (universal bacteria) | Forward primer: GCAGGCCTAACACATGCAAGTC  Reverse primer: CTGCTGCCTCCCGTAGGAGT |

**Supplementary Table 4.** The antibodies used in flow cytometry

|  | Source | Identifier |
| --- | --- | --- |
| PerCP/Cy5.5-CD45 (30-F11) mouse | Biolegend | Cat:103132 RRID: AB_893340 |
| PE anti-mouse MHCII (M5/114.15.2) | Biolegend | Cat:107608 RRID: AB_313323 |
| APC anti-mouse Ly6C (HK1.4) | Biolegend | Cat:128016 RRID: AB_1732076 |
| APC-CD11c (N418) mouse | Biolegend | Cat:117310 RRID: AB_313779 |
| PerCP/Cy5.5-CD11b (M1/70)  mouse | Biolegend | Cat:101227 RRID: AB_893233 |
| PE-CD103 (2E7) mouse | Biolegend | Cat:121405 RRID: AB_535948 |
| PE-PI | Biolegend | Cat: 421301 |
| FITC-Annexin V | Biolegend | Cat: 640914 |

**Supplementary Table 5.** Docking scores among different flagellin and flagellin receptor

|  | DVF | *Salmonella Typhimurium* flagellin | *Escherichia coli* flagellin |
| --- | --- | --- | --- |
| LRRC19 | -628 | 0 | -329 |
| TLR5 | -277 | -732 | -413 |
| NLRC4 | -564 | -1044 | -141 |
